# Supplementary material for: An optimized contact map for GōMartini 3 enabling conformational changes in protein assemblies
Source: Biophys J. 2026 Apr 24;125(11):2695–706. doi: 10.1016/j.bpj.2026.04.024 (PMC13351852; doi:10.1016/j.bpj.2026.04.024)
Supplement: Document S1. Figures S1–S8, Table S1, and supplemental materials and methods [file mmc1.pdf]

Biophysical Journal, Volume *125*

Supplemental information

An optimized contact map for GōMartini 3 enabling conformational changes in protein assemblies

Gustavo E. Olivos-Ramirez, Luis F. Cofas-Vargas, Siewert J. Marrink, and Adolfo B. Poma

Supplementary Material
An optimized contact map for GōMartini 3 enabling conformational changes in protein assemblies

Gustavo E. Olivos-Ramirez[1], Luis F. Cofas-Vargas[1,2], Siewert J. Marrink[3], and Adolfo B. Poma[1,*]

[1]Department of Biosystems and Soft Matter, Institute of Fundamental Technological Research, Polish Academy of Sciences, ul. Pawińskiego 5B, 02-106, Warsaw, Poland

[2]Departamento de Química, Universidad Autónoma Metropolitana-Iztapalapa, Mexico City C.P. 09310, Mexico

[3]Groningen Biomolecular Sciences and Biotechnology Institute, University of Groningen, Nijenborgh 7, 9747 AG Groningen, The Netherlands

[*]E-mail: apoma@ippt.pan.pl

Supplementary figures

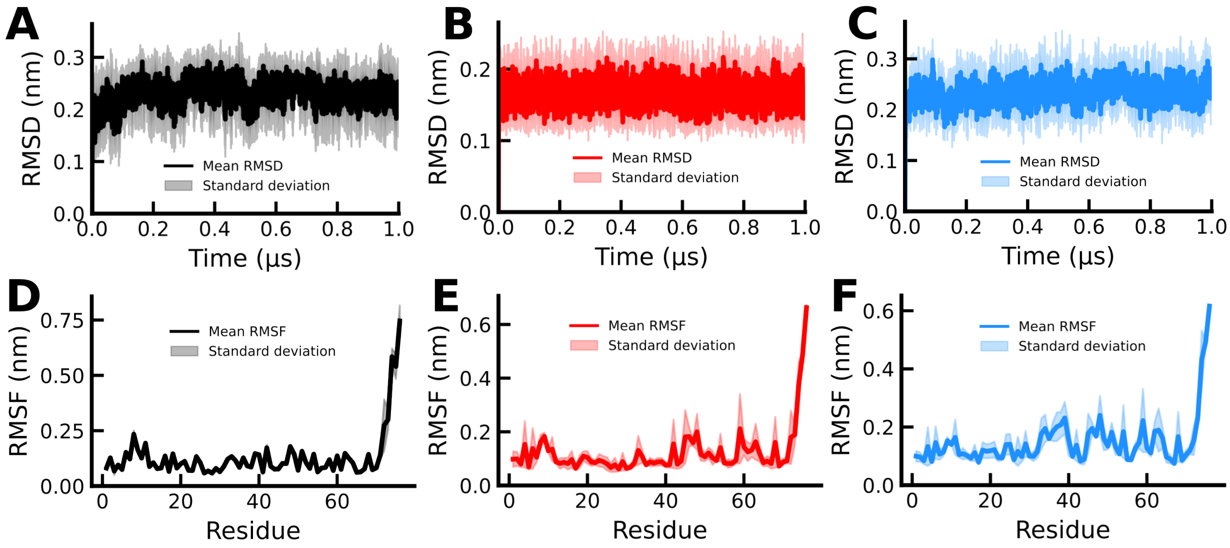

Figure S1: Time evolution of the average RMSD and RMSF of the ubiquitin protein (PDB ID: 1UBQ). Values were averaged from all simulation replicas (R1-R5). Panels A, B, C show the RMSD evolution during 1μ for AA-MD (black), GōMartini 3 with the crystal contact map (red), and GōMartini 3 with the HFC map (sky blue). Panels D, E, and F show the RMSF values for the same system.

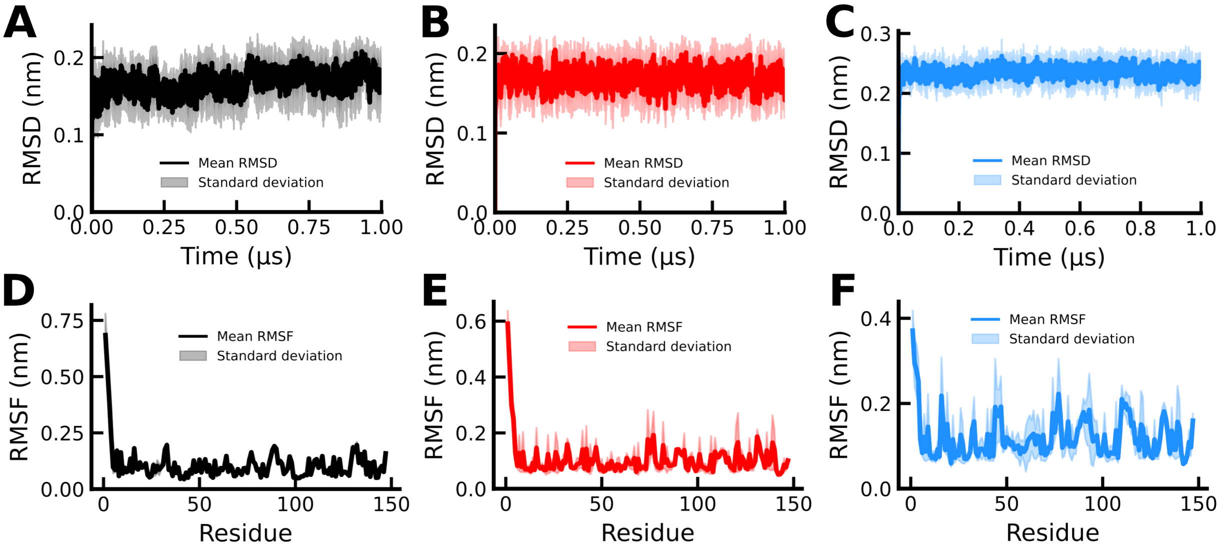

Figure S2: Time evolution of the average RMSD and RMSF of the single cohesin domain (PDB ID: 1AOH). Values were averaged from all simulation replicas (R1-R5). Panels A, B, C show the RMSD evolution during 1μ for AA-MD (black), GōMartini 3 with the crystal contact map (red), and GōMartini 3 with the HFC map (sky blue). Panels D, E, and F show the RMSF values for the same system.

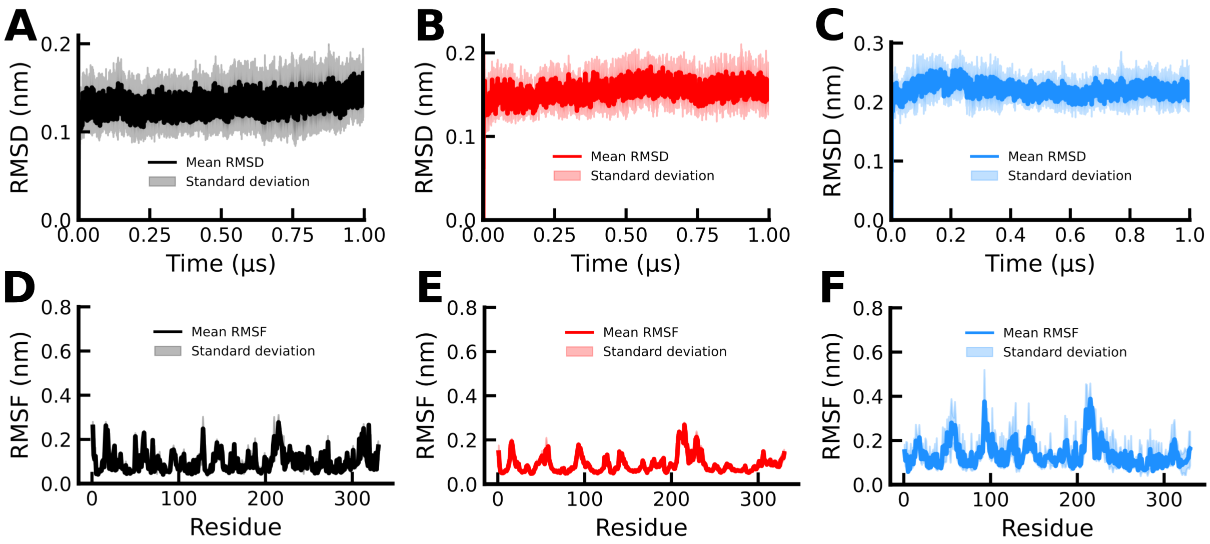

Figure S3: Time evolution of the average RMSD and RMSF of the glycoside hydrolase (PDB ID: 3W0K). Values were averaged from all simulation replicas (R1-R5). Panels A, B, C show the RMSD evolution during 1μ for AA-MD (black), GōMartini 3 with the crystal contact map (red), and GōMartini 3 with the HFC map (sky blue). Panels D, E, and F show the RMSF values for the same system.

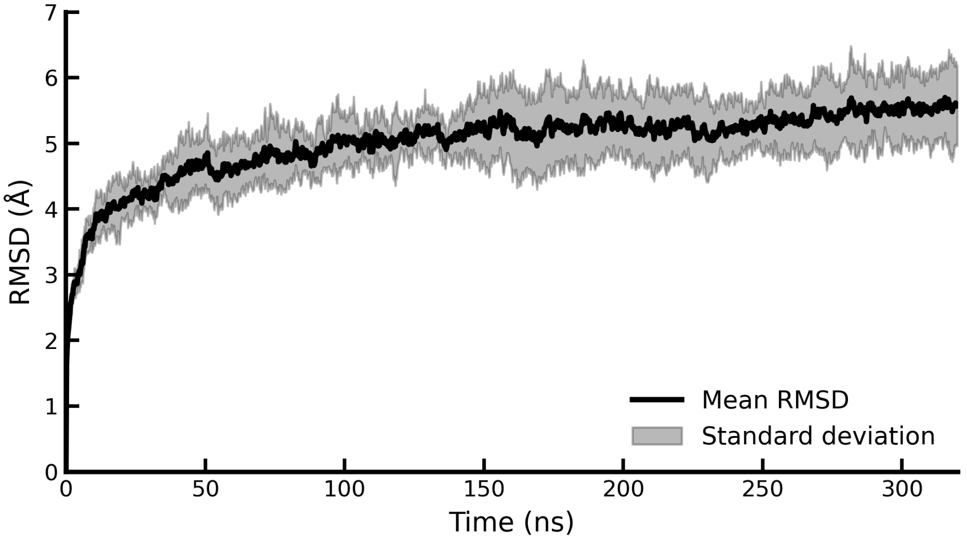

Figure S4: Time evolution of the average RMSD for all AA-MD simulation replicas (R1–R5) of the SARS-CoV-2 S protein.

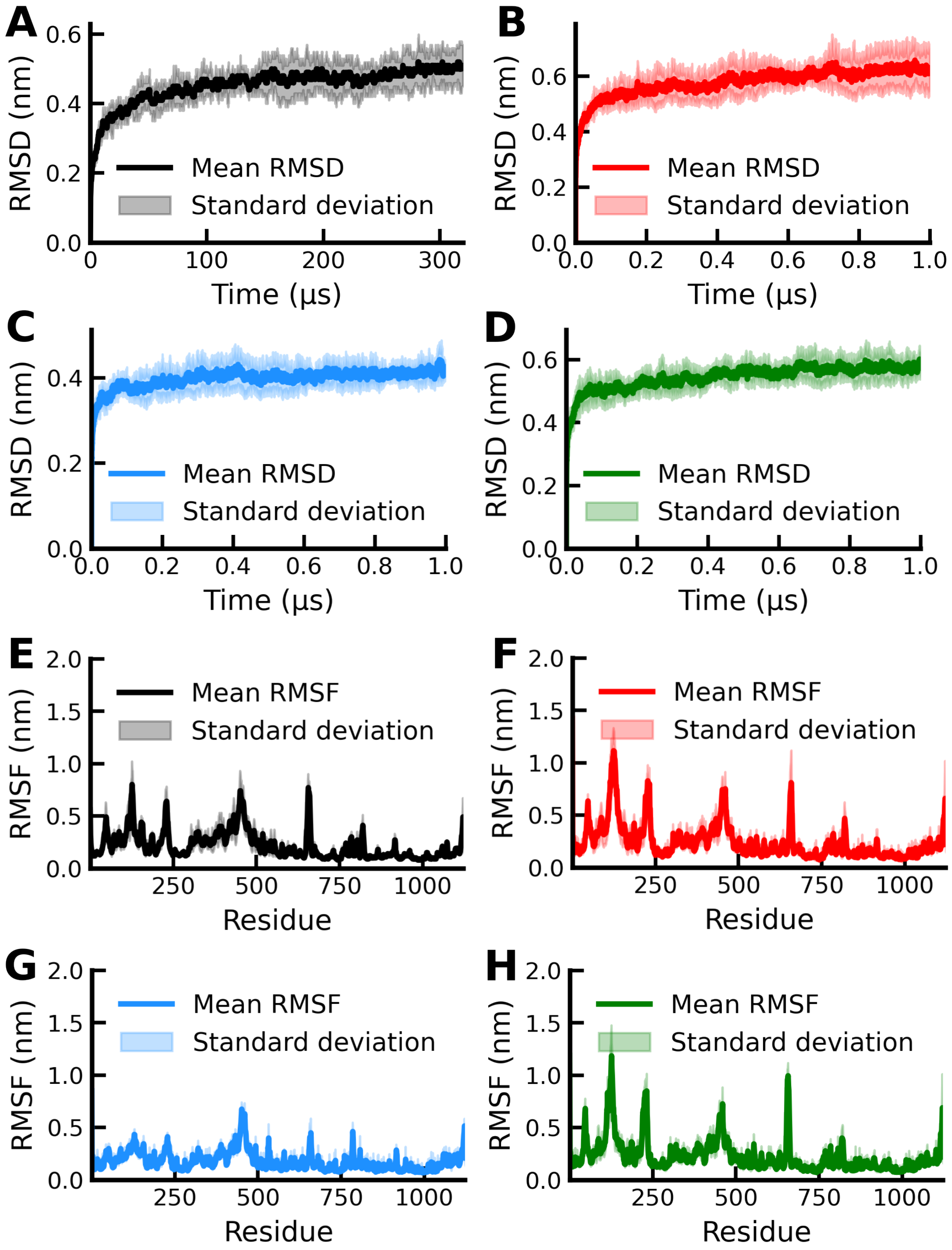

Figure S5: Time evolution of the average RMSD and RMSF for all simulation replicas (R1–R10) of the SARS-CoV-2 S protein (PDB ID: 6VSB), in AA-MD (black), GōMartini optimized-1 (red), optimized-2 (sky blue), and optimized-3 (green).

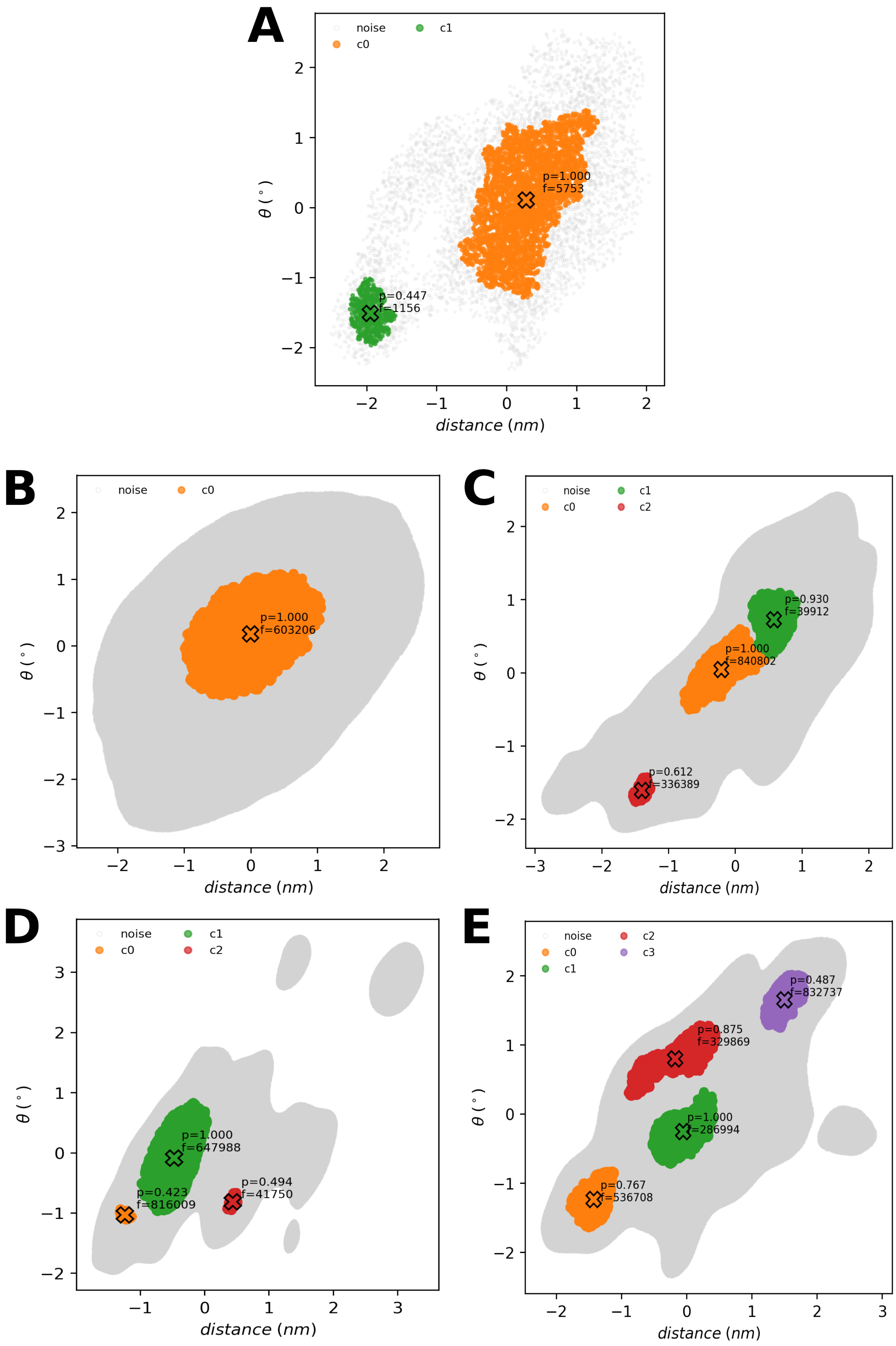

Figure S6: ML-DBSCAN of the most probable conformations of the SARS-CoV-2 S protein based on the CVs used in the FEL, from AA-MD (A), GōMartini 3 Original (B), Optimized-1 (C), Optimized-2 (D), and Optimized-3 (E) simulations.

Supplementary table

Table S1: Summary of the contact information used in each method.

Method	Contact information
AA	None
Original	NC from the initial structure
Optimized-1	HFC interchain + HFC intrachain + HFC that appear during the AA-MD
Optimized-2	HFC interchain + intrachain contacts from the reference structure
Optimized-3	HFC interchain + HFC intrachain

NC = native contacts. HFC = high-frequency contacts.

Convergence analysis of HFC protocol

To provide practical guidance for constructing robust HFC maps, we performed a systematic convergence analysis of different threshold used to define HFC. Thresholds of 0.5, 0.6, 0.7, 0.8, 0.9, and 1 were evaluated every 10 ns (from 90 to 320 ns) using the AA-MD trajectories of the S protein with five independent replicas (Figure S7 and S8). Convergence of the HFC maps was assessed with four descriptors:

(i) **Total HFC count over time.** This quantifies the temporal stabilization of the HFC set by tracking how the total number of HFC evolves as the AA-MD trajectory length increases. The emergence of a plateau indicates convergence.

(ii) **Conserved HFC fraction.** This quantifies how much of the original contact map is preserved in the HFC map by measuring (every 10 ns) the fraction of original contacts that remain present. It is computed as the number of contacts common to both maps divided by the total number of contacts in the original map.

(iii) **Newly acquired HFC.** This quantifies contacts that emerge as the AA-MD simulation progresses and are incorporated into the HFC map but are absent from the original contact map. Therefore, it provides a direct measure of how many new interactions are added beyond those present in the starting structure.

(iv) **Similarity between consecutive HFC maps (Jaccard similarity).** This quantifies how stable the HFC map is over time by comparing the contact sets obtained at consecutive time points using the Jaccard similarity. It is defined as the number of HFC shared by two maps divided by the total number of distinct contacts present in either map. We evaluated this metric from 90 to 320 ns, sampling every 10 ns. Values approaching 1 indicate that consecutive HFC maps are nearly identical, consistent with a stable HFC set.

Threshold choice should meet two important requirements: retaining enough stabilizing

contacts to preserve folding, while removing sufficient contacts from the original map to enable conformational transitions. Permissive thresholds (0.5–0.6) retain a dense network and show higher variability across time and replicas, whereas stringent thresholds (0.9–1) over-prune both conserved and newly formed contacts. Accordingly, intermediate thresholds around 0.7 provide a robust compromise, yielding reproducible contact networks (Figures S7–S8). Consistently, a stress tests at the extreme thresholds (0.5 and 1), using 1 us of CG-MD, showed that the cutoff = 1 increased RMSD relative to both cutoff = 0.5 and the AA reference. RMSF also increased globally, indicating reduced structural stability. Meanwhile, the cutoff = 0.5 stayed closer to the AA benchmark but may hinder conformational transitions due to its denser contact network (Figure S9).

Overall, this convergence workflow provides practical guidance for defining HFC maps from AA-MD trajectories and motivates the default threshold used here (0.7). As a guideline, we recommend using AA-MD segments exceeding 100 ns after initial relaxation and verifying convergence prior to HFC extraction with the same four metrics. For large multimeric assemblies, multiple independent replicas improve reproducibility and enable direct quantification of inter-replica variability. While the optimal threshold may be system-dependent, we consider a value of 0.7 to provide a robust starting point based on the observed balance between stability and conformational flexibility.

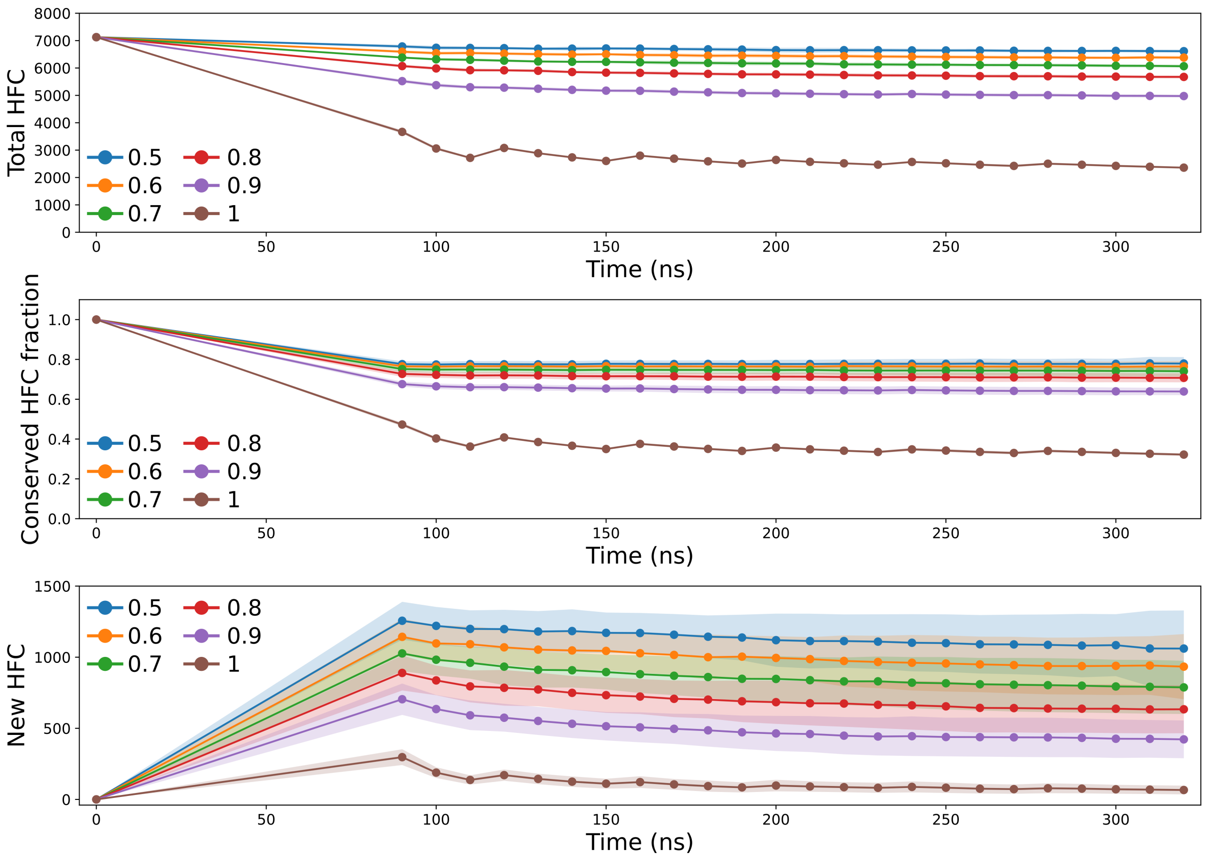

Figure S7: Convergence descriptors for HFC maps of the SARS-CoV-2 S protein computed across persistence thresholds (0.5–1) over the 90–320 ns window (10-ns stride). (A) Total number of HFC. (B) Conserved HFC ratio relative to the original contact map at $t = 0$ ns. (C) Number of newly formed HFC not present in the original map. Solid lines show the mean of each cutoff and shaded areas the SD across replicas (see Methods for definitions).

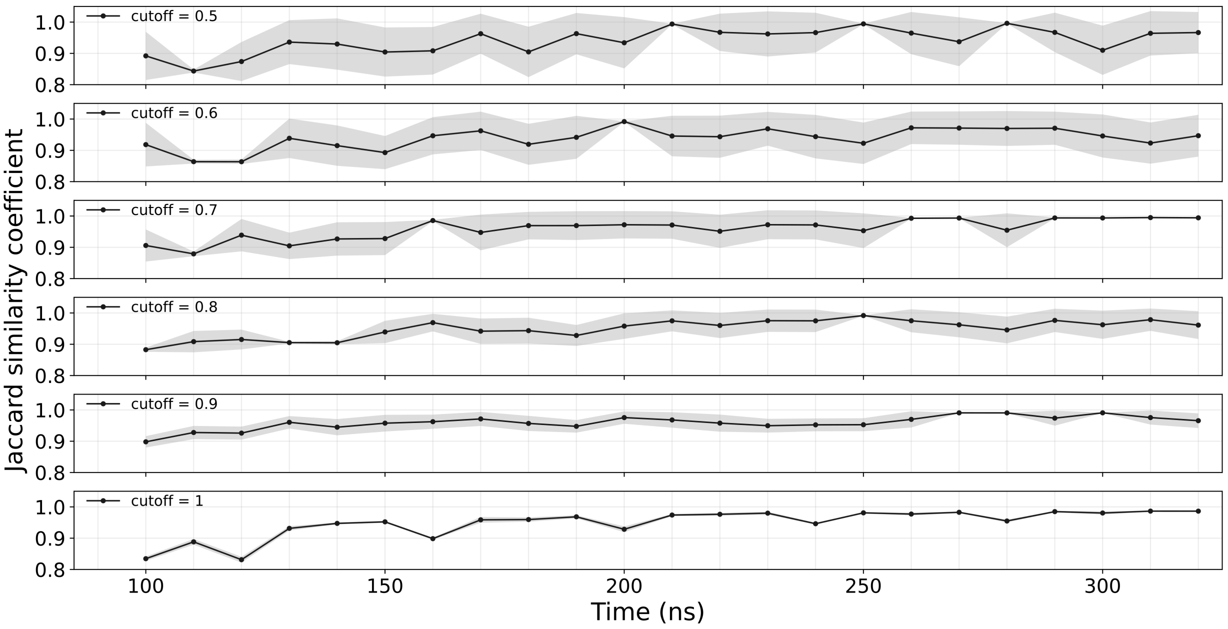

Figure S8: Jaccard similarity between consecutive HFC maps (every 10-ns) for persistence thresholds of 0.5–1 over the 90–320 ns window. Curves show the mean and shaded areas the SD across AA-MD replicas (see Methods for definition).

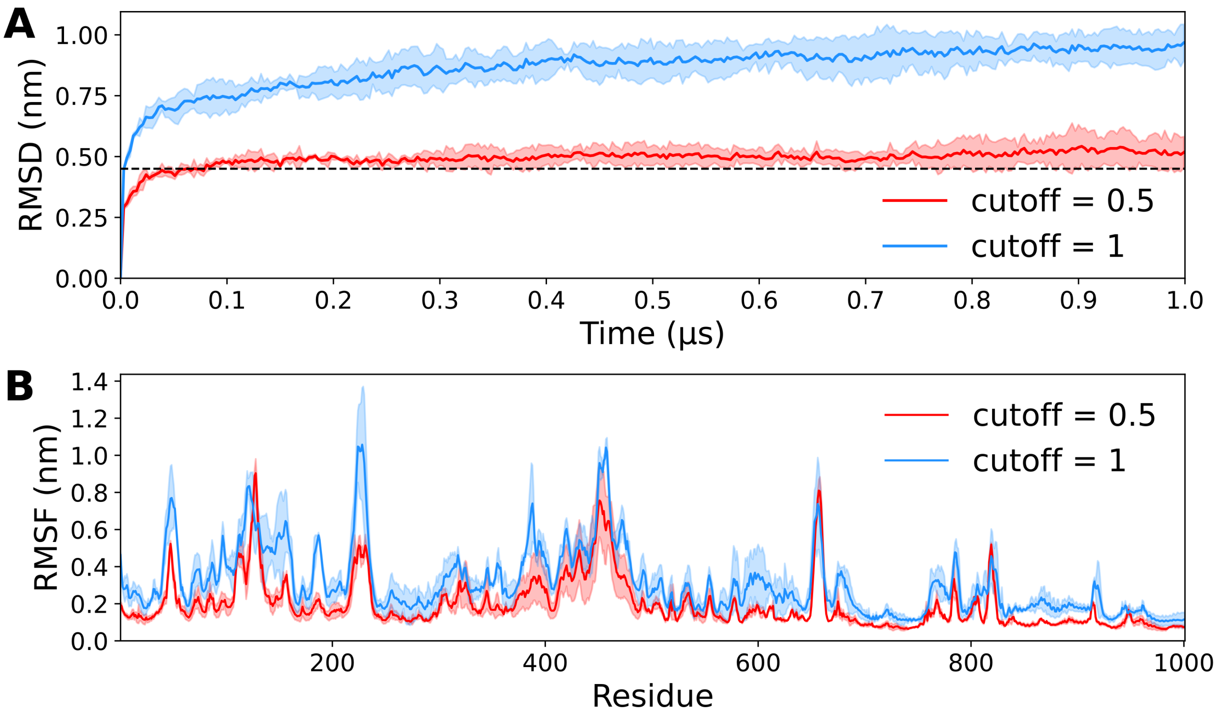

Figure S9: CG-MD simulation at extreme persistence thresholds (stress tests) for the SARS-CoV-2 S protein. (A) RMSD over 1 μs for HFC maps defined at cutoff = 0.5 and 1 (mean $\pm$ SD across three replicas); dashed line indicates the AA-MD mean RMSD. (B) Per-residue RMSF for the same conditions.
