# Supplementary material for: An optimized contact map for GōMartini 3 enabling conformational changes in protein assemblies
Source: Biophys J. 2026 Apr 24;125(11):2695–706. doi: 10.1016/j.bpj.2026.04.024 (PMC13351852; doi:10.1016/j.bpj.2026.04.024)
Supplement: Document S2. Article plus supplemental information [file mmc3.pdf]

Biophysical Journal
Article

Biophysical Society

An optimized contact map for GōMartini 3 enabling conformational changes in protein assemblies

Gustavo E. Olivos-Ramirez,[1] Luis F. Cofas-Vargas,[1,2] Siewert J. Marrink,[3] and Adolfo B. Poma[1,*]
[1]Biosystems and Soft Matter Division, Institute of Fundamental Technological Research, Polish Academy of Sciences, ul. Pawińskiego 5B, 02-106, Warsaw, Poland; [2]Departamento de Química, Universidad Autónoma Metropolitana-Iztapalapa, Mexico City, Mexico; and [3]Groningen Biomolecular Sciences and Biotechnology Institute, University of Groningen, Groningen, the Netherlands

ABSTRACT Advances in structural biology, particularly cryo-electron microscopy, have enabled high-resolution characterization of complex protein assemblies. These developments underscore the need for computational approaches capable of describing biologically relevant conformational changes over extended timescales. GōMartini 3 is a coarse-grained approach that demonstrates computational efficiency and versatility across several systems, from membrane-binding proteins and soluble proteins to intrinsically disordered proteins, while preserving key physicochemical features. In this work, we introduce an optimized approach that integrates dynamic contact information from all-atom molecular dynamics (AA-MD) simulations to refine the contact map in GōMartini simulations and select the AA-MD structure consistent with the refined map. Specifically, we define high-frequency contacts, which reduce the number of original Gō contact set by ≈20%–30%, thereby improving the representation of conformational states beyond the original approach in Martini 3. Benchmarking different contact selection criteria revealed that including intra- and interchain high-frequency contacts in protein assemblies captures structural flexibility and domain dynamics. The method was tested on single-chain globular proteins and on the SARS-CoV-2 spike protein. Overall, the optimized contact map improves sampling efficiency and expands the accessible conformational landscape. The full framework is available as an open-source tool for large-scale simulations of protein assemblies.

SIGNIFICANCE Large protein assemblies often rely on conformational rearrangements to regulate their function. However, capturing these motions with all-atom molecular dynamics (MD) simulations remains computationally challenging. Coarse-grained models offer an alternative, but they frequently over-stabilize native structures, limiting access to biologically relevant states. Here, we introduce a generalizable strategy that integrates dynamic information from all-atom MD simulations to identify high-frequency contacts and refine the GōMartini 3 approach. This optimization reduces redundant constraints while preserving structural stability, enabling broader and more efficient sampling of the conformational space. The open-source framework enhances the applicability of coarse-grained simulations for studying the dynamics of multidomain and multimeric protein assemblies.

INTRODUCTION

With the advancement of experimental techniques such as cryo-electron microscopy, it is now possible to solve structures of increasingly complex biological systems, often in the megadalton (MDa) range.[1] Experimental structures provide static or ensemble snapshots, but molecular dynamics (MD) simulations are essential to reveal the continuous conformational transitions underlying biological function. All-atom (AA) MD simulations provide atomic-level detail; however, their computational cost limits accessible system sizes and timescales, typically reaching only from nanoseconds to a few microseconds. Performing MD simulations at the millisecond timescale[2] or with very large systems[3–6] requires extensive high-performance computing resources, which are not always accessible. To overcome these limitations, coarse-grained (CG) approaches have emerged as an alternative, offering significant reductions in computational cost while enabling the exploration of large systems and longer timescales.[7]

Submitted November 28, 2025, and accepted for publication April 20, 2026.
*Correspondence: apoma@ippt.pan.pl
Editor: Yi Qin Gao.
https://doi.org/10.1016/j.bpj.2026.04.024
© 2026 The Authors. Published by Elsevier Inc. on behalf of Biophysical Society.
This is an open access article under the CC BY license (http://creativecommons.org/licenses/by/4.0/).

CG models reduce molecular complexity by mapping groups of atoms onto interaction sites or beads, thereby smoothing the energy landscape while maintaining a molecular-level representation.[8] This simplification accelerates simulations by two to three orders of magnitude compared to atomistic models,[9] allowing the observation of rare events such as folding, binding, and large-scale conformational transitions. Among CG frameworks, the Martini force field[10] is one of the most widely used. Initially developed for lipid membranes, it has since evolved into a general-purpose model applicable to proteins, nucleic acids, and large supramolecular complexes, enabling simulations that reach the millisecond regime.[11–14] The latest Martini 3 release includes several approaches aimed at stabilizing protein structures: the elastic network (EN) to maintain structural integrity,[15] the GōMartini 3 model that integrates native contact (NC) information for balanced flexibility,[16,17] and the OLIVES multiscale pipeline, which uses hydrogen-bonding information to stabilize the structure.[18] Moreover, a recent approach termed OliGōmers[19] has been designed for multimer systems to study oligomerization and aggregation phenomena.

The GōMartini 3 framework is widely used to study single proteins and protein complexes, providing a balance between structural stability and conformational flexibility.[20–22] This approach integrates Martini 3 nonbonded interactions with a Gō-like[23,24] potential defined over NCs derived from a reference structure. The native contact map (CM) is typically constructed using geometric overlap (OV) and repulsive contacts of structural units (rCSU) criteria,[25] and Lennard-Jones (LJ) potentials are applied to stabilize the corresponding tertiary and quaternary interactions during simulations. However, because the CM is derived from a single static model (i.e., a crystal structure), the resulting potential confines the system within one free-energy basin, restricting transitions between alternative conformational states. This limitation becomes especially critical for large or multichain complexes, where fixed NCs may artificially constrain inter-domain motions or slow down conformational changes.[17] The switching[26] and multiple-basin[27] GōMartini variants partially address this issue by interpolating between CMs derived from two known states, yet they require the availability of experimentally solved conformations for both. An alternative perturbation-based optimization approach (*PoG*ō) was shown to fine-tune the ϵ and σ parameters in the Gō potentials using AA-MD data, thereby capturing essential dynamics comparable to AA-MD.[17,28] For systems where only one structure is available, it remains challenging to model long-timescale conformational changes.

Multiscale modeling integrates two or more simulation approaches to bridge different spatial and temporal resolutions.[29] In biomolecular systems, such methods connect atomistic accuracy with CG efficiency, enabling the study of processes that span from nanometers to hundreds of nanometers and from nanoseconds to milliseconds. Multiscale approaches can generally be divided into two categories: hybrid (parallel) and sequential (serial).[30,31] In hybrid schemes, multiple levels of resolution coexist within a single simulation, for example, quantum mechanics/molecular mechanics (QM/MM) or MM/CG, while sequential approaches use information from higher-resolution models to parameterize or guide lower-resolution representations. By combining the structural detail of atomistic simulations with the efficiency of CG models, multiscale strategies provide improved physical realism, access to extended timescales, and a substantial reduction in computational cost.[32] In this context, AA-MD simulations on the nanosecond-to-microsecond scale[33] can be used to refine GōMartini 3 contact definitions, yielding a data-driven model that retains structural stability while enhancing conformational sampling. However, to achieve this integration, a dedicated framework is required to translate atomistic dynamic information accurately into the CG representation. This strategy benefits from and aligns with the findability, accessibility, interoperability, and reusability (FAIR) principles,[34] as it promotes the reuse of existing data to improve the accuracy of lower-resolution models.

In this study, we present a framework to optimize the CM for GōMartini 3, with the main objective of enhancing conformational sampling in large protein assemblies. Our approach (Figure 1) reduces the number of Gō contacts by constructing a refined CM that includes only high-frequency contacts (HFC) derived from AA-MD simulations. We validated the HFC concept using three single-chain soluble proteins and the SARS-CoV-2 spike (S) protein. Overall, the optimized GōMartini 3 model substantially improves conformational sampling by reducing the number of Gō contacts, producing a more flexible yet stable CG model capable of exploring functionally relevant states consistent with Martini 3 across extended timescales.

MATERIALS AND METHODS

AA-MD simulations

To assess the HFC within the GōMartini 3 approach, we first evaluated the accuracy of the optimization using single-chain globular proteins: ubiquitin, the single cohesin domain, and the glycoside hydrolase (comprising 76, 147, and 330 amino acids, respectively). The structures of these proteins were retrieved from the PDB[35] (PDB ID: 1UBQ, 1AOH, and 3W0K, respectively). The proteins were repaired using PDBFixer.[36] Missing atoms were added, and protonation was performed at pH 7.4. AA-MD simulations were conducted in GROMACS 2023.5.[37] Each system was placed in a cubic box with 10 Å of padding. TIP3P water molecules were used for solvation. The system was neutralized by adding sodium (Na^+) and chloride (Cl^-) counterions at a concentration of 0.15 M. Energy minimization was performed using the steepest descent algorithm[38] for

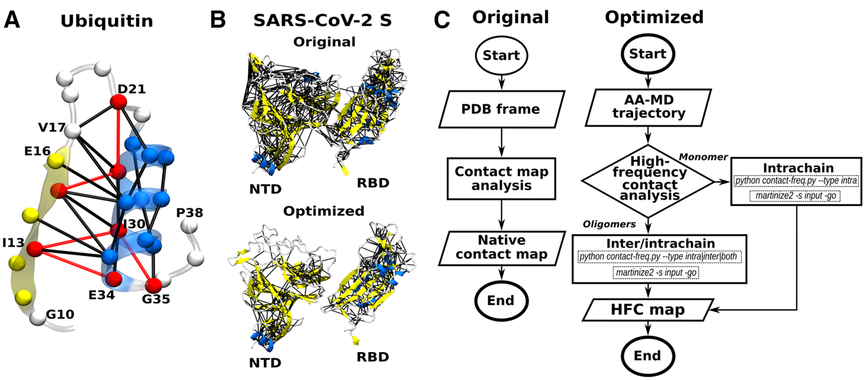

Figure 1. Refinement of HFC for GōMartini 3 simulations. (A) Residues 11–37 of ubiquitin. This image displays HFC (black) and low-frequency contacts (red) derived from 1 μs atomistic MD sampling. HFC are defined as residue pairs persisting in at least 70% of simulation frames. (B) Contacts between the receptor-binding domain (RBD) and the N-terminal domain (NTD) of the SARS-CoV-2 S protein (PDB ID: 6VSB). The top image shows native contacts from the crystal structure, while the bottom image displays HFC extracted from 320 ns of atomistic MD simulations across five replicas. (C) Comparison between the original contact map algorithm (left) and the optimized version using HFC (right). For a Figure360 author presentation of Figure 1, see https://doi.org/10.1016/j.bpj.2026.04.024#mmc2.

50,000 steps. Equilibration was performed in two stages: NVT (100 ps) and NPT (200 ps). Temperature was equilibrated at 300 K using the V-rescale thermostat,[39] while pressure was equilibrated at 1 bar using the Parrinello-Rahman barostat.[40] During minimization and equilibration, harmonic restraints of 1,000 kJ $mol^{-1}nm^{-2}$ were applied to the Cα atoms in their Cartesian coordinates. The linear constraint solver (LINCS) algorithm[41] was used to constrain all bonds, and long-range electrostatics were treated with the particle-mesh Ewald (PME) method.[42] Production simulations were conducted for 1 μs with five independent replicas in the NPT ensemble, using an integration time step of 2 fs with the leap-frog integrator.[43]

In addition, we studied the case of the wild-type (WT) S protein from SARS-CoV-2. Atomistic trajectories of the WT S protein with a single receptor-binding domain (RBD)-up were obtained from a previous work.[44] In that study, the WT structure (PDB ID: 6VSB) was retrieved from the PDB. MD simulations were performed using the Amber ff14SB force field.[45] The system was placed in a box with padding of 12 Å and solvated using the TIP3P water model.[46] Sodium and chloride ions were added to neutralize the system at a concentration of 0.150 M. Energy minimization was conducted using the steepest descent algorithm with 2,000 steps, followed by conjugate gradient equilibration with 3,000 steps. Temperature equilibration was performed in two steps: NVT heating from 0 to 100 K over 50 ps, followed by NPT heating from 100 to 300 K over 100 ps. Harmonic restraints of 10 kcal mol^{-1} $Å^{-2}$ were applied during the minimization and equilibration steps. These restraints were gradually reduced from 10 to 0.1 kcal mol^{-1} $Å^{-2}$ through a short 6 ns simulation at 300 K. Pressure equilibration was performed using the Monte Carlo (MC) barostat[47] at 1 atm. Temperature control was achieved with the Langevin thermostat[48] using a collision frequency of 1 ps^{-1}. Hydrogen mass repartitioning (HMR) was applied to the systems to achieve integration time steps of 4 fs.[49] Production was performed for 320 ns with five replicas (1.6 μs in total). Based on the root-mean-square deviation (RMSD) (Figure S4), we determined that this system stabilizes after 80 ns. For this reason, in the HFC analysis, we used only the last 240 ns of each replicate. Finally, we concatenated all trajectories (6,000 frames in total, 1,200 per trajectory) with a frame-saving interval of 200 ps (0.2 ns) per frame.

CM determination protocol

To explore the conformational space of the protein systems, we performed CG simulations using the GōMartini 3 approach.[11,16,17] This requires defining a CM, which is generated through a combination of the OV and rCSU methods.[25] The CM is computed from an atomistic structure, and the resulting contacts are incorporated into the GōMartini 3 simulations as LJ potentials. This approach is widely adopted and has become a standard in CG simulations.[17] To improve the capabilities of this methodology, a careful selection of the optimal configuration of contacts was made. For this, we tested optimized CMs based on a selection of the HFC. Figure 1C shows the step-by-step protocol developed to enhance the GōMartini 3 approach, and Table S1 presents the definition of each optimized CM.

The first step involved converting each frame (from the AA-MD simulations) to PDB format, as the script used for CM calculation only supports this file type. A contact was defined when the distance between Cα atoms of those residues was within 3–11 Å. This distance range follows the contact definition employed in the GōMartini 3 framework, where NCs are determined using a 3–11 Å cutoff to capture both short- and longer-range interactions. The same criterion is applied after contact identification (via atomic OVs and rCSU methods) to construct the Gō potentials and ensure consistency with the CM definition implemented in *martinize2*.[50] Frequencies were calculated for each pair of contacts across all frames. Only contacts with a frequency $\geq$ 0.7 were selected. HFC were identified for both intra- and interchain interactions. After determining HFC, a reference frame was selected for GōMartini 3

simulations. The selected frame was the one containing the highest number of HFC, obtained by comparison between the list of HFC and the contacts of each individual frame. In addition, if a contact is classified as high frequency but not present in the reference structure (due to longer distances), the average distance of those pairs over the trajectory was calculated and then used to compute the $r_{\min}$ values and define the virtual sites. Finally, using the *martinize2* tool, new topology files (ITP files and CG structure) were generated, keeping the list of HFC, while the original contacts from the reference frame (i.e., low-frequency contacts) were removed.

All these steps were automated using a custom pipeline built around two Python scripts designed to streamline the identification and classification of HFC and the generation of input files. These scripts allowed us to identify HFC efficiently and build topologies for subsequent GōMartini 3 simulations. The first script, termed *traj_to_pdb.py*, splits a trajectory into individual PDB frames at user-defined intervals, controlled by the –stride parameter. During this step, the chain index ranges of interest must be specified with the –range parameter (i.e., 1–1,121, 1,122–2,242, and 2,243–3,364 for the S protein). The second script, termed *contact_freq.py*, processes each frame to determine HFC and classifies them as interchain, intrachain, or both, depending on the –type argument. Using these criteria, we constructed the corresponding CM for the three optimized models. When using –type both, the optional –all-hf flag can be enabled to include HFC that are present along the trajectory into the selected reference frame. Additionally, the –go-eps parameter allows customization of the ε value used in the Gō potential (default: 9.414 kJ/mol). For multichain systems, chain IDs are specified using the –merge option. A contact-frequency threshold is set via the –threshold parameter, commonly defined as 0.7, meaning the contact must be present in at least 70% of the frames. Selection of the HFC threshold was assessed through a systematic convergence analysis considering six threshold values (0.5–1.0; see supplemental information). Based on this analysis, the following parameters were adopted: five replicas, 320 ns of AA-MD trajectory per replica, and sub-sampling of frames every 200 ps for HFC evaluation. Convergence was observed at approximately 200 ns; therefore, only the last 240 ns of each trajectory were considered for the final HFC analysis. If DSSP[51] is not available in the system path, the path to the `mkdssp` executable must be explicitly defined using the –dssp option. Once the reference frame containing the highest number of HFC is identified, the corresponding CM is passed to *martinize2* to generate GōMartini 3 topology files.

For single-chain globular proteins, we evaluated only HFC of the intrachain type, as each system comprised a single protomer. For the case of the S protein, we evaluated three different CM configurations[1]: a first optimized CM including all HFC (5,408), both inter- and intrachain contacts of the reference structure, and additional ones that are not found in that structure but are mapped along the AA trajectory[2]; a second optimized CM including only interchain HFC, while the intrachain part was treated as nonhigh frequency (6,973). Similarly, the reference frame containing HFC was selected.[3] A third optimized CM included only HFC of the inter- and intrachain space (4,966), using the reference frame containing HFC as well. Since HFC were used in 1–3, these were referred to as optimized-1, -2, and -3, respectively. All the scripts used in this protocol are available at https://github.com/GoMartini3-tools/ContactFreq/tree/main, and it includes a Jupyter notebook. The interaction energy of all GōMartini 3 simulations was set to 15.0 kJ mol^{-1}.

GōMartini 3 simulations

Once each optimized set of NCs was defined, GōMartini 3 simulations were performed with GROMACS 2023.5[37] using the Martini 3 force field.[11] The system topology was built using the *martinize2* algorithm,[50] and secondary structure was assigned using DSSP v.3.1.4. The initial structure was subjected to vacuum energy minimization for 5,000 steps using the steepest descent algorithm. The system was then solvated in a cubic box of 10 Å of padding for the single-chain globular proteins and in a 12 × 12× 25 nm^3 box for the S protein, using Martini water beads. The systems were neutralized by adding sodium and chloride ions to reach a concentration of 0.15 M. A second energy minimization step was performed for the solvated and neutralized system for 5,000 steps. During equilibration, position restraints were applied to backbone beads (BBs). Equilibration was carried out in two stages. The first stage was performed in the NVT ensemble at 300 K using the V-rescale thermostat,[39] with a coupling time of 1.0 ps. This phase lasted 5 ns with integration steps of 20 fs. The second equilibration step was carried out in the NPT ensemble at 1 atm using the C-rescale thermostat,[52] with isotropic pressure coupling = 10^{-4} bar^{-1} and a pressure coupling time constant of 12 ps. This phase lasted 10 ns, and it also used 20 fs integration time. Production simulations were conducted in the NPT ensemble. Five replicas of 1 μs each (totaling 5 μs) were initially performed for all systems. Then, for the S protein, we conducted a 10 μs MD simulation with 10 replicas (cumulative 100 μs) for each optimized protocol and for the original CM.

Structural stability analysis

To evaluate the structural stability and residue-level flexibility of all AA-MD and GōMartini 3 simulations, we calculated the RMSD and the root-mean-square fluctuation (RMSF) for each system. The Hellinger distance (H) was then used to quantify the similarity between the RMSF distributions of each optimized model against the AA-MD.[53] This method is a well-defined statistical metric for assessing the similarity between two probability distributions.[53]

For discretely sampled MD data, the Hellinger distance between two normalized RMSF distributions $p = \{p_i\}$ and $q = \{q_i\}$ is computed as

$$H(p,q) = \frac{1}{\sqrt{2}}\left(\sum_{i=1}^{N}\left(\sqrt{p_i} - \sqrt{q_i}\right)^2\right)^{1/2} \quad (1)$$

where p_i and q_i denote the normalized histogram of the residue-specific RMSF distributions. This metric satisfies $0 \le H \le 1$, where $H = 0$ indicates identical distributions and $H = 1$ corresponds to no OV.

The Hellinger distance measures the OV between fluctuation profiles, providing a robust indicator of whether two simulations explore similar conformational space. Because RMSF distributions reflect the breadth and shape of local free-energy basins, lower Hellinger values indicate more similar flexibility patterns and preservation of residue-level dynamics. Empirically, values below 0.3 denote excellent agreement, while those up to approximately 0.7 still reflect acceptable similarity.

Identification of metastable states

To identify the metastable states of the SARS-CoV-2 S protein, we first constructed its conformational landscape using two well-established collective variables (CVs).[54] Metastable states were then extracted using the multilevel density-based spatial clustering of applications with noise (ML-DBSCAN) algorithm,[55] an extension of the classical DBSCAN method.[56] Density-based clustering assumes that MD trajectories sample conformations according to the Boltzmann distribution, such that regions of high sampling density correspond to low-free-energy basins (metastable states). For a given neighborhood radius (ϵ), DBSCAN defines the local ϵ neighborhood of a conformation x_i as

$$\mathcal{N}_\epsilon(x_i) = \{ x_j : d(x_i, x_j) \le \epsilon \} \quad (2)$$

and classifies x_i as a core point when $|\mathcal{N}_\epsilon(x_i)| \ge$ min_samples. Clusters emerge as density-connected sets of core points, allowing the identification of arbitrarily shaped free-energy basins without assuming linear separability or Gaussianity.

ML-DBSCAN extends this concept by performing DBSCAN across multiple density levels, using a decreasing sequence of neighborhood radii ($\epsilon_1 > \epsilon_2 > \cdots > \epsilon_n$). Coarse levels capture broader basins, whereas finer levels resolve sub-basins within them. The resulting clusters are integrated into a hierarchical structure by evaluating the parent-child OV between successive levels. A child cluster C_c is assigned to a parent C_p when

$$\text{persistence} = \frac{|C_c \cap C_p|}{|C_c|} \ge 0.7 \quad (3)$$

Clusters persisting across levels represent stable metastable states in the underlying free-energy landscape (FEL). In our analysis, we first discarded low-probability CV points (<0.1 in the FEL). All CVs were Z scored prior to clustering. ML-DBSCAN was then applied across a sequence of radii (ϵ = 0.045–0.030) with a fixed minimum neighborhood size (min_samples = 600). Only clusters with persistence $\ge$ 0.7 and population $\ge$ 1500 conformation were retained. Computations were performed using a GPU-accelerated implementation based on RAPIDS cuML v.25,[57] enabling efficient processing of the > 10^6 conformations from the 10 replicas (10 μs dataset).

RESULTS AND DISCUSSION

In Figure 1, we introduce the concept of HFC used for enhancing GōMartini 3 CMs. In the original workflow (Figure 1C), contacts are mapped from the crystal structure using the OV + rCSU concepts over residue pairs (i, $|i - j| \ge 3$), resulting in a CM that preserves the folded topology. However, in protein assemblies, the large surface area between protomers can generate excessive definitions of Gō contacts. This effect can overconstrain the system to a single energetic basin, limiting the conformational sampling. To address this limitation, our approach selectively corrects both inter- and intrachain contacts to reduce redundancy and enhance sampling. Specifically, we refine the CM by removing low-frequency contacts identified from AA-MD. As shown in Figure 1A, high-frequency (*black*) and low-frequency (*red*) contacts are overlaid for ubiquitin. The removal of low-frequency contacts, such as the hydrophobic pair I13–I30 (*red lines* in Figure 1A), still preserves the integrity of the hydrophobic core. This refinement becomes even more critical in complex systems such as the SARS-CoV-2 S protein. As shown in Figure 1B, the contact network illustrates the differences between the original and enhanced CM. By pruning low-frequency contacts, the CG model gains the flexibility required to explore multiple conformational states.

For validation, we benchmarked HFC in three single-chain globular proteins (ubiquitin, cohesin domain, and glycoside hydrolase) and evaluated their stability against AA-MD simulations. Afterward, using the SARS-CoV-2 S protein as a case study, we generated three distinct HFC maps for GōMartini 3 simulations (termed as optimized-1, -2, and -3) with the objective of determining the effect of intra- and interchain contact. Our method quantifies both intra- and interchain contacts throughout the trajectory, defining an HFC as any contact with a frequency $\ge$ 0.7. In addition, it identifies and selects the frame containing the highest number of HFC. In optimized-1, both inter- and intrachain HFC defined in the selected frame, together with additional HFC identified throughout the AA-MD trajectory, were included (NCs = 5,408). In optimized-2, the interchain HFC identified in the selected frame were incorporated while preserving the existing intrachain contacts from that same frame (NCs = 6,973). Finally, in optimized-3, both inter- and intrachain HFC (NCs =

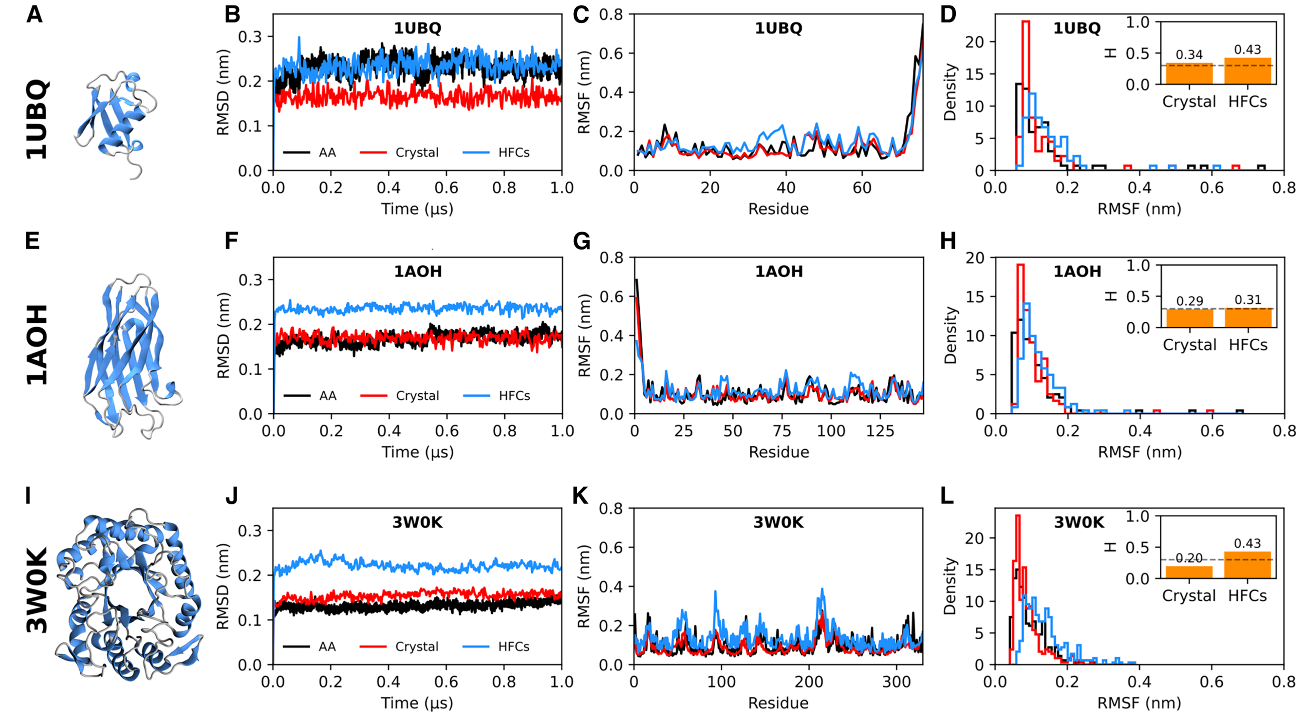

Figure 2. Flexibility of single-chain globular proteins in AA and CG simulations. Structural stability of 1UBQ, 1AOH, and 3W0K was evaluated using 1 µs atomistic MD and GōMartini 3 simulations (ε = 15.0 kJ/mol). (A) Ribbon representation of PDB IDs: 1UBQ; (B)–(D) RMSD, RMSF, and Hellinger distance (H) comparisons for PDB: 1UBQ. (E) Ribbon representation of PDB: 1AOH; (F)–(H) RMSD, RMSF, and Hellinger distance (H) comparisons for PDB: 1AOH. (I) Ribbon representation of PDB: 3W0K; (J)–(L) RMSD, RMSF, and Hellinger distance (H) comparisons for PDB: 3W0K. Comparisons are shown between crystal-based and HFC-enhanced models against the atomistic reference. Dashed lines denote the critical H threshold for distribution agreement.

4,966) were incorporated (see materials and methods and Table S1). We demonstrate that both intra- and interchain HFC enable the observation of significant conformational transitions, in contrast to the traditional approach. This implementation enables the study of large macromolecular assemblies with a more accurate and dynamic definition of NCs, preserving tertiary and quaternary structure while facilitating efficient exploration of long-timescale conformational dynamics.

CM optimization in single-chain globular proteins

We first validated the HFC refinement protocol using single-chain proteins. AA-MD simulations of 1 µs each (five replicas per system) were performed for ubiquitin,[58] the cohesin domain,[59] and glycoside hydrolase. HFC were extracted from these trajectories to construct refined CMs, which were subsequently employed in GōMartini 3 simulations of equivalent length and replica count. Conventional GōMartini 3 simulations using crystal-derived CMs were performed in parallel as controls. Compared to the crystal-derived CM, the refined HFC maps retained 80.4%, 72.1%, and 75.0% of contacts for 1UBQ, 1AOH, and 3W0K, respectively. This corresponds to an approximate 20%–28% reduction in contact count relative to the standard protocol, yielding a less constrained model expected to better capture intrinsic protein flexibility.

To evaluate the effect of removing low-frequency contacts on model accuracy, we compared the RMSD and RMSF profiles obtained from AA-MD, the standard GōMartini 3 model based on the crystal-derived CM, and the enhanced GōMartini 3 model incorporating HFC. For ubiquitin (Figure 2A), the RMSD profile of the HFC model overlapped the AA-MD reference, while the RMSF profiles remained consistent (Figure 2B). Only residues 30–40 and 44–46 exhibited slightly increased flexibility (≈1.3 Å). RMSF distributions largely overlapped the atomistic reference, with an $H = 0.34$ for the crystal and $H = 0.43$ for the HFC (Figures 2C and 2D). For the cohesin domain (Figure 2E), the HFC model showed a small RMSD increase of ≈1.0 Å (Figure 2F) but achieved excellent agreement in flexibility (Figure 2G), with $H = 0.29$ for the crystal and $H = 0.31$ for the HFC model (Figure 2H). In the glycoside hydrolase (Figure 2I), the HFC produced a moderate RMSD increase of ≈1.5 Å (Figure 2J) while maintaining similar RMSF patterns (Figure 2K), with distributional OV slightly favoring the crystal ($H = 0.20$) over the HFC ($H = 0.43$) (Figure 2L). Overall, the removal of low-frequency contacts still preserved structural stability and flexibility across all three systems. Nevertheless, for single-chain globular

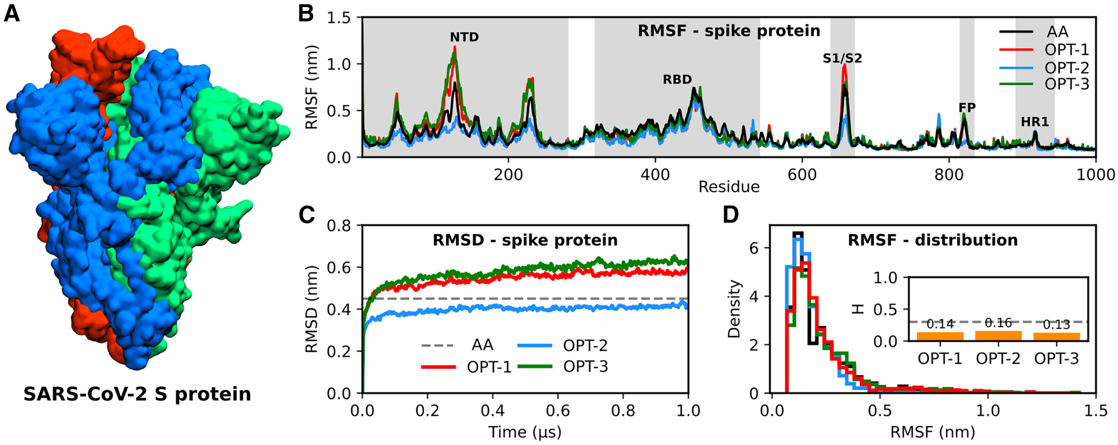

Figure 3. Flexibility of the SARS-CoV-2 S protein in optimized GōMartini 3 simulations. (A) Trimeric S protein stability evaluated from 1 μs simulations (five replicas) at $\epsilon = 15.0$ kJ/mol. (B) RMSF profiles for the RBD-up conformation comparing atomistic and optimized models, with shaded regions marking functional domains. (C) RMSD profiles for each model; the black line indicates the atomistic reference average (0.45 nm) from 320 ns of sampling. (D) RMSF distributions and Hellinger distance (H) values quantifying similarity to the reference, with dashed lines denoting the critical H threshold.

proteins, the standard GōMartini 3 model remains slightly more consistent with AA-MD. Individual profiles for those systems are found in Figures S1–S3.

CM optimization in protein assemblies

For protein assemblies, such as the ≈0.6 MDa trimeric SARS-CoV-2 S protein (Figure 3A), we evaluated the contribution of inter- and intrachain HFC to the stability and dynamics. Three HFC optimizations (optimized-1, -2, and -3) were constructed and validated against AA-MD simulations. These comparative setups (Table S1) were designed to identify the optimization scheme that best preserves structural stability while providing sufficient flexibility to enhance conformational sampling.

A detailed analysis of the principal domains (RBD, N-terminal domain [NTD], S1/S2, FP, and HR1) in the chain with the RBD-up revealed similar flexibility patterns for optimized-1 and -3 (Figure 3B). The RMSF profiles of these two models showed enhanced mobility in the NTD, with optimized-3 most closely resembling the reference AA model. The RMSF of the RBD remained generally consistent across all models, while optimized-3 better reproduced the reference behavior in the S1/S2 and HR1 regions. The RMSF of FP was also more accurately captured by optimized-3. For optimized-2, all domains displayed lower RMSF values, reflecting a more rigid behavior. Overall, these RMSF profiles indicate that optimized-1 and -3 preserve the dynamic features of the AA model. The RMSD analysis further supported these observations (Figure 3C). All three optimized models reached RMSD convergence; however, optimized-2 exhibited lower values, indicative of reduced conformational exploration due to its stiffer intrachain network. Optimized-1 and -3, in turn, presented similar RMSD profiles, slightly higher than the reference model (*dashed gray line* in Figure 3C) but differing by only ≈0.1 nm. Moreover, H values between the RMSF distributions remained below 0.3 across all models (Figure 3D), confirming good agreement with the reference. Minor variations in H originated from local differences within the main domains (i.e., RBD and NTD), highlighted in the RMSF profiles as shadows. These results demonstrate that incorporating inter- and intrachain HFC preserves the flexibility of the GōMartini 3 models relative to the AA-MD simulation. Individual profiles for the S protein can be found in Figures S4 and S5.

Application in a large-scale protein system

To further evaluate the robustness of the enhanced GōMartini 3 model, we extended the S protein simulations to a timescale of 10 μs (10 replicas). Since the initial structure contained one RBD-up, we selected standard CVs to characterize its conformational fluctuations within the sampled space. Specifically, we measured the distance (nm) between the centers of mass of residues I338–W442 and Q512–G532 from the RBD-up and its opposite RBD, corresponding to the direction of the up-to-down transition, as well as the angular fluctuation (θ) of the RBD relative to a vertical vector defined by the HR1 domain. These CVs were used here to construct the FEL and to compute the probability distribution of metastable states, as shown in Figure 4. Details of all metastable states identified by ML-DBSCAN are presented in Figure S6.

The AA model was characterized by two well-defined minima within the conformational space of the RBD-up. However, S_1 concentrates most of the probability, with the second state presenting low probability ($p < 0.5$) (Figure 4A). Although the AA-MD simulation was relatively short (320 ns), the RBD domain exhibited some flexibility, within the range of 4.0–5.5 nm of distance variation and angle fluctuation in the range of $-7°$ and $-4°$. In contrast, the

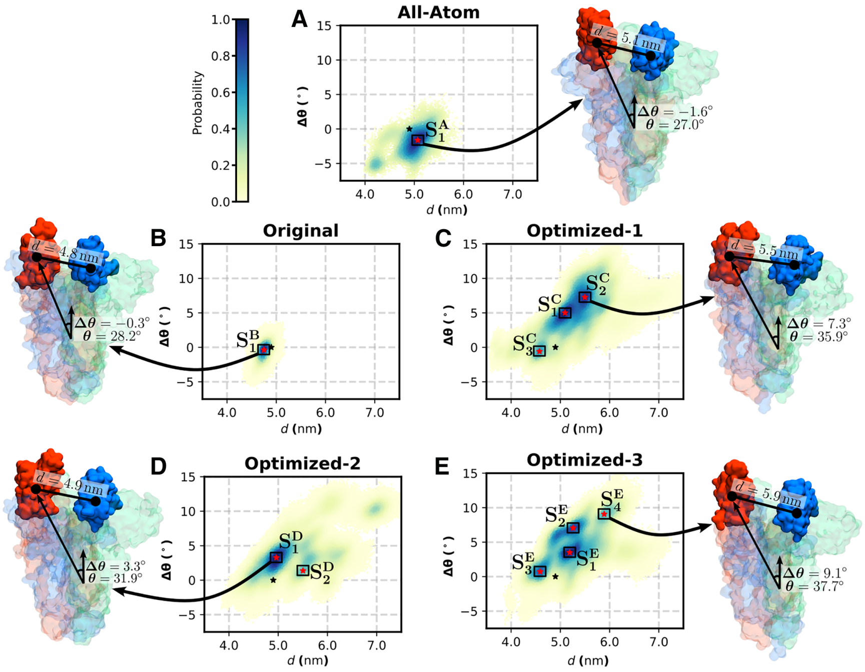

Figure 4. Probability states of the WT SARS-CoV-2 S protein in 10 μs GōMartini 3 simulations ($\epsilon = 15.0$ kJ/mol). (A and B) Comparison of optimized CM configurations (1, 2, and 3) against the atomistic (AA) reference and standard GōMartini 3. (C–E) Optimized models incorporating HFC (frequency > 70%). Red stars indicate independent clusters (S_n), while black stars mark the reference AA structure. Only clusters with $\geq$ 50% probability are shown, with snapshots highlighting the most diverse centroids relative to the AA model. Distance (d) and $\Delta\theta$ are reported based on FEL ($\Delta\theta = \theta - \theta_0$, where $\theta_0 = 28.6°$).

standard GōMartini 3 simulation (original) sampled a narrower conformational space, exhibiting a single dominant state (Figure 4B). This FEL was notably more confined, despite the extended simulation time and multiple replicas employed. Here, the RBD remained close to its original conformation (*black star*). The limited sampling capacity of the original model originates from an excessive number of Gō contacts within the RBDs, NTDs, and HR1 domains. This effect is especially relevant in multimeric assemblies, where interfacial complexity increases the number of contacts and, consequently, the structural constraints that hinder conformational transitions.

In the three optimized models, the sampling space was expanded (Figures 4C–4E). In these cases, the distance between the RBDs changed approximately from 3.6 to 7.5 nm, and the angle fluctuated between −4.9° and 15° (Figures 4C–4E). Although all optimized models enhanced the sampling capacity, their main differences arose from the distinct probabilities assigned to each metastable state (Table 1). Optimized-1 presented three metastable states, optimized-2 presented two states, and optimized-3 presented four states, all of them with $p > 0.5$. Notably, optimized-2 concentrated the density in S_1 and S_2, while other minima were observed but with lower probabilities ($p < 0.5$). This suggests that optimizing only the interchain contacts is insufficient to improve the model, as intrachain interactions also play a key role in maintaining flexibility and enabling conformational transitions (Figure 4D). Meanwhile, optimized-1 and -3 presented higher probabilities distributed among three and four states, respectively, with similar coverage in the CV space. However, optimized-3 outperformed the other models by exhibiting multiple high-probability states ($p > 0.5$) spanning different distances and angles (Figure 4E). This optimization relied on fewer stabilizing contacts but still improved conformational sampling while preserving structural stability.

To further evaluate the model, we calculated the number of interchain contacts in each state. This analysis was performed for the highest-probability states and for the conformations closest to the initial state. The initial state is defined as S_0 (*black stars* in Figure 4). Although S_0 was not a high-probability state in simulations with HFC, it was included because it provides insights into contact reorganization when the system explores those coordinates. For this purpose, all conformations (frames) within the CV range of the initial structure (RBD distance = 4.92 nm and angle = 0.0°, with a standard deviation of ±0.01) and of the high-probability metastable states were included in the analysis. To determine Gō contacts, the selected frames were backmapped using the one-bead-per-residue approach.[60] The total number of interchain contacts in the S trimer, as well as those specifically associated with the RBD-up, are summarized in Table 1.

The total number of contacts differed substantially among all GōMartini 3 simulations (ranging from 6,772 to 6,893) compared with the AA-MD simulation (7,441 ± 46) in the S_0 state. These discrepancies arise from the Gō potential and the Martini 3 force field, both of which aim to preserve structural stability. As a result, although the systems explore similar regions of the CV space, the overall assembly diverges in specific structural regions, leading to variations in the contact network. This

Table 1. Number of interchain contacts in the metastable states for the optimized models

Model	CM	States	p	Total NCs	ΔNCs	RBD NCs	Identity (%)
All atom	–	S_0	<0.01	7,144 ± 46	–	40 ± 5	–
Original	7,289	S_0	–	6,772 ± 45	↓372	49 ± 7	33.8
Optimized-1	5,408	S_0	–	6,893 ± 47	↓251	50 ± 12	21.5
Optimized-2	6,973	S_0	–	6,838 ± 72	↓251	55 ± 10	29.2
Optimized-3	4,966	S_0	–	6,888 ± 81	↓256	59 ± 14	30.8
All atom	–	S_1^A	1.0	7,131 ± 50	–	39 ± 8	–
Original	7,289	S_1^B	1.0	6,774 ± 49	↓515	52 ± 7	80.5
Optimized-1	5,408	S_1^C	1.0	6,891 ± 51	↑1,483	45 ± 12	–
		S_2^C	0.93	6,757 ± 58	↑1,349	55 ± 8	–
		S_3^C	0.61	6,860 ± 62	↑1,452	38 ± 10	–
Optimized-2	6,973	S_1^D	1.0	6,757 ± 58	↓216	32 ± 15	–
		S_2^D	0.50	6,802 ± 57	↓171	19 ± 5	–
Optimized-3	4,966	S_1^E	1.0	6,917 ± 78	↑1,951	52 ± 13	–
		S_2^E	0.88	6,904 ± 50	↑1,938	42 ± 11	–
		S_3^E	0.78	6,861 ± 48	↑1,895	59 ± 10	–
		S_4^E	0.50	6,885 ± 56	↑1,919	62 ± 17	–

Different superscript letters in S_n (A–E) indicate the corresponding model: A, all atom; B, original; C, optimized-1; D, optimized-2; and E, optimized-3. CM, the number of Gō contacts in each contact map; p, probability; ΔNCs, difference in contacts with respect to AA for S_0 and to the CM for the different states (S_n); identity, number of contacts equal to the AA for S_0 and to the CM for the different states (S_n).

effect is particularly evident in the RBDs, where the total number of contacts remains comparable across models, yet their identities differ markedly (Table 1). The OV of RBD contact identities ranged from 21.5% to 33.8%. Theoretically, a highly accurate model should reproduce not only similar conformations but also similar contact identities. However, as previously discussed, the distinct configurations of each optimized model likely constrain conformational changes at specific sites, preventing identical contact rearrangements. An additional explanation may be that other CVs, beyond those used here, could better capture transitions and improve consistency of contact identity among optimized models.

Besides, the metastable states observed in the optimized models displayed distinct contact profiles. In optimized-1 and -3, the total number of contacts increased markedly, even though their CMs were constructed exclusively from HFC (Table 1). In contrast, optimized-2 exhibited metastable states (S_1^D and S_2^D) with contact counts similar to those of the initial CM, indicating limited structural reorganization. Although the number of RBD contacts remained within the standard deviation across models, the reduced number of states and their associated probabilities suggest that optimized-2 was the least effective configuration. Optimized-1 showed improved conformational sampling with three distinct metastable states, yet optimized-3 further enhanced sampling and stability, displaying up to four high-probability states. In optimized-3, the total number of contacts increased substantially relative to the original CM. The minima S_1^E, S_2^E, S_3^E, and S_4^E exhibited an average increase of ≈1,900 total contacts and elevated RBD contact counts of 52 ± 13, 42 ± 11, 59 ± 10, and 62 ± 10, respectively. These states occupied regions of the CV space with larger angle $(>5^\circ)$ and distance (>5 nm) values than the initial configuration (Figure 4E). Notably, S_4^E corresponds to a more extended RBD-up conformation, consistent with experimentally observed structures (PDB ID: 7SBL and 7UPY), underscoring the accuracy of this optimization. Overall, these findings demonstrate that optimized-3, despite employing the smallest amount of contact information (only 4,966 HFC), consistently produced the largest number of high-probability minima. This indicates that the model effectively recovers key stabilizing contacts while achieving superior conformational sampling efficiency.

Further work is required to optimize the atomistic simulation time necessary to determine HFC. Since this study aims to develop an improved methodology for sampling systems in the MDa range, AA simulations for such large assemblies may still represent a major limitation. Our results indicate that incorporating only a few hundred nanoseconds from multiple independent AA-MD replicates can enhance sampling efficiency. It is also worth noting that our work recycled trajectories from previously published simulations. In this way, the reuse of existing atomistic information is promoted as a practical strategy to improve CG models in specific cases, reducing both computational cost and data redundancy. Moreover, our current approach has focused exclusively on reducing the number of contacts defined in the CM. Recent work by Kalutskii et al.[28] has instead concentrated on refining the LJ potentials. In this sense, both concepts could be combined to improve not only the accuracy but also the sampling efficiency of CG simulations. Additionally, the implementation of a Gō-like network with potentials dependent on distance fluctuations extracted from AA-MD simulations may represent another promising route to optimizing the GōMartini 3 model.

CONCLUSION

This work presents an approach to optimize the CM and enhance the GōMartini 3 framework. We introduce the concept of HFC for the simulation of large protein assemblies. Structural and stability analyses (i.e., RMSD and RMSF) performed on single-chain globular proteins demonstrate that HFC preserves the native structure within the same range as the classical approach, which uses the experimental structure as a reference to build the GōMartini protein model. The classical method yields a closer description of the native state, with limited fluctuations outside it, closely resembling the behavior of the reference AA model.

In protein assemblies, such as the SARS-CoV-2 S protein, the inclusion of HFC significantly improved conformational sampling around the native state. By benchmarking different optimized CMs, we found that incorporating both inter- and intrachain HFC resulted in a broader exploration of the conformational landscape. This global optimization of NCs was the most efficient strategy in terms of Gō contact information, enabling the sampling of a larger number of conformational states (up to four) with a minimal set of contacts. Moreover, these states were more evenly distributed across the conformational space.

Our implementation is particularly valuable when a wide exploration of the conformational landscape is required, such as for processes occurring on the millisecond timescale. Additionally, reducing the number of Gō contacts while still retaining the native state of protein assemblies provides a more realistic biophysical description of their response to mechanical forces or temperature perturbations. Our work can boost other recent implementations, such as Switching Gō-Martini and Multiple-Basin Gō-Martini implementations,[26,27] to explore the vast protein conformational space between different metastable states (active and inactive). Similarly, our work can be combined with *PoGō* (an optimization of GōMartini 3 LJ parameters[28] for capturing the protein's essential dynamics).

The CM optimization protocol we developed uses the *martinize2* tool and delivers new parameters compatible with GōMartini 3. This work may help identify previously inaccessible conformational states that underlie the chemical or biological functionality of complex biomolecular assemblies in Martini 3.

Finally, the applicability of the HFC refinement is expected to be most relevant for large, multimeric assemblies with extensive interfacial contact networks, where classical crystal-derived CMs may overconstrain conformational transitions and limit conformational sampling. This CM refinement may facilitate the identification of previously inaccessible conformations relevant to biological function and mechanistic studies. The HFC approach is particularly suitable for moderately large protein systems for which at least 200 ns of AA-MD simulations are computationally feasible, as this duration is necessary to obtain a converged HFC analysis. Moreover, intrinsically disordered regions (IDRs) are typically not well captured by HFC definitions due to their dynamic nature; in such cases, Martini3-IDP[61] or dedicated disordered-protein parametrizations may be more appropriate.

DATA AVAILABILITY

All input files, simulation data, and processed results generated and analyzed in this study have been deposited in the Zenodo repository under https://doi.org/10.5281/zenodo.16023028 (or https://zenodo.org/records/16023028) and will be publicly available upon publication.

ACKNOWLEDGMENTS

A.B.P. acknowledges earlier discussions on the topic with Rodrigo A. Moreira and financial support from the National Science Center, Poland, under grant 2022/45/B/NZ1/02519. This work thanks the Polish high-performance computing infrastructure PLGrid (HPC Center: ACK Cyfronet AGH) for providing computer facilities and support within computational grant nos. PLG/2024/017332 and PLG/2025/018510.

AUTHOR CONTRIBUTIONS

G.E.O.-R. and L.F.C.-V. contributed equally to this work. G.E.O.-R. performed the MD simulations, contributed to the algorithm development, analyzed and validated the data, and drafted the manuscript. L.F.C.-V. developed the ContactFreq algorithm, performed MD simulations, and contributed to manuscript revision. S.J.M. participated in discussions and provided critical feedback. A.B.P. conceived the study, supervised the work, contributed to the algorithm discussion, acquired funding, and revised the manuscript. All authors have given approval to the final version of the manuscript.

DECLARATION OF INTERESTS

The authors declare no competing interests.

SUPPLEMENTAL INFORMATION

Supplemental information can be found online at https://doi.org/10.1016/j.bpj.2026.04.024.

REFERENCES

1. Bai, X., G. McMullan, and S. H. W. Scheres. 2015. How cryo-EM is revolutionizing structural biology. *Trends Biochem. Sci.* 40:49–57. https://www.sciencedirect.com/science/article/pii/S096800041400187X.
2. Lindorff-Larsen, K., S. Piana, …, D. E. Shaw. 2011. How fast-folding proteins fold. *Science.* 334:517–520. https://doi.org/10.1126/science.1208351.
3. Dommer, A., L. Casalino, …, R. E. Amaro. 2022. #COVIDisAirborne: AI-enabled multiscale computational microscopy of delta SARS-CoV-2 in a respiratory aerosol. *Int. J. High Perform. Comput. Appl.* 37:28–44. https://doi.org/10.1177/10943420221128233.
4. Perilla, J. R., and K. Schulten. 2017. Physical properties of the HIV-1 capsid from all-atom molecular dynamics simulations. *Nat. Commun.* 8:15959. https://doi.org/10.1038/ncomms15959.
5. Farafonov, V. S., M. Stich, and D. A. Nerukh. 2023. Complete Virion Simulated: All-Atom Model of an MS2 Bacteriophage with Native

Genome. *J. Chem. Theor. Comput.* 19:7924–7933. https://doi.org/10.1021/acs.jctc.3c00846.

6. Sanbonmatsu, K. Y., S. Joseph, and C. Tung. 2005. Simulating movement of tRNA into the ribosome during decoding. *Proc. Natl. Acad. Sci. USA.* 102:15854–15859. https://doi.org/10.1073/pnas.0503456102.
7. Poma, A. B., A. H. Caldas, and L. M. Sandonas. 2025. Recent advances in machine learning and coarse-grained potentials for biomolecular simulations. *Biophys. J.* 125:327–343.
8. Monticelli, L., S. K. Kandasamy, ..., S.-J. Marrink. 2008. The Martini Coarse-Grained Force Field: Extension to Proteins. *J. Chem. Theor. Comput.* 4:819–834. https://doi.org/10.1021/ct700324x.
9. Fitzgerald, G., J. DeJoannis, and M. Meunier. 2015. 1 - Multiscale modeling of nanomaterials: recent developments and future prospects. *In* Modeling, Characterization, and Production of Nanomaterials. V. K. Tewary and Y. Zhang, eds Woodhead Publishing, Woodhead Publishing Series in Electronic and Optical Materials, pp. 3–53. https://www.sciencedirect.com/science/article/pii/B9781782422280000016.
10. Marrink, S. J., H. J. Risselada, ..., A. H. de Vries. 2007. The MARTINI Force Field: Coarse Grained Model for Biomolecular Simulations. *J. Phys. Chem. B.* 111:7812–7824. https://doi.org/10.1021/jp071097f.
11. Souza, P. C. T., R. Alessandri, ..., S. J. Marrink. 2021. Martini 3: a general purpose force field for coarse-grained molecular dynamics. *Nat. Methods.* 18:382–388. https://doi.org/10.1038/s41592-021-01098-3.
12. Risselada, H. J. 2021. Martini 3: a coarse-grained force field with an eye for atomic detail. *Nat. Methods.* 18:342–343. https://doi.org/10.1038/s41592-021-01111-9.
13. Kjølbye, L. R., G. P. Pereira, ..., P. C. Souza. 2022. Towards design of drugs and delivery systems with the Martini coarse-grained model. *QRB Discov.* 3:e19. https://www.cambridge.org/core/product/BF2AA08FACA5AF1980FD683EC4BF1517.
14. Kharche, S., M. Yadav, ..., D. Sengupta. 2024. Improved Protein Dynamics and Hydration in the Martini3 Coarse-Grain Model. *J. Chem. Inf. Model.* 64:837–850. https://doi.org/10.1021/acs.jcim.3c00802.
15. Périole, X., M. Cavalli, ..., M. Ceruso. 2009. Combining an Elastic Network with a Coarse-Grained Molecular Force Field: Structure, Dynamics, and Intermolecular Recognition. *J. Chem. Theor. Comput.* 5:2531–2543. https://doi.org/10.1021/ct9002114.
16. Poma, A. B., M. Cieplak, and P. E. Theodorakis. 2017. Combining the Martini and structure-based coarse-grained approaches for molecular dynamics studies of conformational transitions in proteins. *J. Chem. Theor. Comput.* 13:1366–1374. https://doi.org/10.1021/acs.jctc.6b00986.
17. Souza, P. C. T., L. Borges-Araújo, ..., S. Thallmair. 2025. GōMartini 3: From large conformational changes in proteins to environmental bias corrections. *Nat. Commun.* 16:4051. https://doi.org/10.1038/s41467-025-58719-0.
18. Pedersen, K. B., L. Borges-Araújo, ..., B. Schiøtt. 2024. OLIVES: A Gō-like Model for Stabilizing Protein Structure via Hydrogen Bonding Native Contacts in the Martini 3 Coarse-Grained Force Field. *J. Chem. Theor. Comput.* 20:8049–8070. https://doi.org/10.1021/acs.jctc.4c00553.
19. Korshunova, K., J. Kiuru, ..., B. M. H. Bruininks. 2024. Martini 3 OliGōomers: A Scalable Approach for Multimers and Fibrils in GROMACS. *J. Chem. Theor. Comput.* 20:7635–7645. https://doi.org/10.1021/acs.jctc.4c00677.
20. Cofas-Vargas, L. F., G. E. Olivos-Ramírez, ..., A. B. Poma. 2024. Nanomechanical Footprint of SARS-CoV-2 Variants in Complex with a Potent Nanobody by Molecular Simulations. *Nanoscale.* 16:18824–18834. https://doi.org/10.1039/D4NR02074J.
21. Duve, T., L. Wang, ..., S. Thallmair. 2025. Martini 3 Protein Models - A Practical Introduction to Different Structure Bias Models and their Comparison. Preprint at bioRxiv. https://doi.org/10.1101/2025.03.17.643608.
22. Koehler, M., A. Ray, ..., D. Alsteens. 2021. Molecular insights into receptor binding energetics and neutralization of SARS-CoV-2 variants. *Nat. Commun.* 12:6977. https://doi.org/10.1038/s41467-021-27325-1.
23. Cofas-Vargas, L. F., R. A. Moreira, ..., A. B. Poma. 2024. The Go-Martini Approach: Revisiting the concept of contact maps and the modelling of protein complexes. *Acta Phys. Pol. A.* 145:S9. https://appol.ifpan.edu.pl/index.php/appa/article/view/145_S9.
24. Poma, A. B., T. T. M. Thu, ..., M. S. Li. 2021. Nanomechanical stability of Aβ tetramers and fibril-like structures: Molecular dynamics simulations. *J. Phys. Chem. B.* 125:7628–7637.
25. Wołek, K., A. Gómez-Sicilia, and M. Cieplak. 2015. Determination of contact maps in proteins: A combination of structural and chemical approaches. *J. Chem. Phys.* 143:243105. https://doi.org/10.1063/1.4929599.
26. Yang, S., and C. Song. 2024. Switching Gō-Martini for Investigating Protein Conformational Transitions and Associated Protein–Lipid Interactions. *J. Chem. Theor. Comput.* 20:2618–2629. https://doi.org/10.1021/acs.jctc.3c01222.
27. Yang, S., and C. Song. 2025. Multiple-Basin Gō-Martini for Investigating Conformational Transitions and Environmental Interactions of Proteins. *J. Chem. Theor. Comput.* 21:5304–5321. https://doi.org/10.1021/acs.jctc.5c00256.
28. Kalutskii, M., C. J. Wilson, ..., M. Igaev. 2026. Improving Conformational Ensembles of Folded Proteins in GoōMartini. *J. Chem. Theor. Comput.* https://doi.org/10.1021/acs.jctc.5c01816.
29. Lin, K., and Z. Wang. 2023. Multiscale Mechanics and Molecular Dynamics Simulations of the Durability of Fiber-Reinforced Polymer Composites. *Commun. Mater.* 4:66. https://doi.org/10.1038/s43246-023-00391-2.
30. Ayton, G. S., W. G. Noid, and G. A. Voth. 2007. Multiscale modeling of biomolecular systems: In serial and in parallel. *Curr. Opin. Struct. Biol.* 17:192–198. https://www.sciencedirect.com/science/article/pii/S0959440X07000346.
31. Kmiecik, S., D. Gront, ..., A. Kolinski. 2016. Coarse-Grained Protein Models and Their Applications. *Chem. Rev.* 116:7898–7936. https://doi.org/10.1021/acs.chemrev.6b00163.
32. Jagger, B. R., S. E. Kochanek, ..., A. J. Mulholland. 2020. Multiscale simulation approaches to modeling drug–protein binding. *Curr. Opin. Struct. Biol.* 61:213–221. https://www.sciencedirect.com/science/article/pii/S0959440X20300142.
33. Mokhtari, O., E. Bignon, and Y. Karami. 2026. DynaRepo: the repository of macromolecular conformational dynamics. *Nucleic Acids Res.* 54:D393–D401. https://doi.org/10.1093/nar/gkaf1130.
34. Amaro, R. E., J. Åqvist, ..., M. Orozco. 2025. The need to implement FAIR principles in biomolecular simulations. *Nat. Methods.* 22:641–645. https://doi.org/10.1038/s41592-025-02635-0.
35. Berman, H. M. 2000. The Protein Data Bank. *Nucleic Acids Res.* 28:235–242. https://doi.org/10.1093/nar/28.1.235.
36. Eastman, P., J. Swails, ..., V. S. Pande. 2017. OpenMM 7: Rapid development of high performance algorithms for molecular dynamics. *PLoS Comput. Biol.* 13:e1005659. https://doi.org/10.1371/journal.pcbi.1005659.
37. Abraham, M., T. J. Murtola, ..., E. Lindahl. 2015. Gromacs: high performance molecular simulations through multi-level parallelism from laptops to supercomputers. *SoftwareX.* 1–2:19–25. https://www.sciencedirect.com/science/article/pii/S2352711015000059.
38. Cauchy, A.-L. 1847. Méthode générale pour la résolution des systèmes d'équations simultanées. *C. R. Acad. Sci.* 25:536–538. https://cs.uwaterloo.ca/y328yu/classics/cauchy-en.pdf.
39. Bussi, G., D. Donadio, and M. Parrinello. 2007. Canonical sampling through velocity rescaling. *J. Chem. Phys.* 126:014101. https://doi.org/10.1063/1.2408420.
40. Parrinello, M., and A. Rahman. 1981. Polymorphic transitions in single crystals: A new molecular dynamics method. *J. Appl. Phys.* 52:7182–7190. https://doi.org/10.1063/1.328693.
41. Hess, B., H. Bekker, ..., J. G. E. M. Fraaije. 1997. LINCS: A linear constraint solver for molecular simulations. *J. Comput. Chem.* 18:1463–1472. https://onlinelibrary.wiley.com/doi/abs/10.1002/%28SICI%291096-987X%28199709%2918%3A12%3C1463%3A%3AAID-JCC4%3E3.0.CO%3B2-H.

42. Petersen, H. G. 1995. Accuracy and efficiency of the particle mesh Ewald method. *J. Chem. Phys.* 103:3668–3679. https://doi.org/10.1063/1.470043.

43. Van Gunsteren, W. F., and H. J. C. Berendsen. 1988. A Leap-Frog Algorithm for Stochastic Dynamics. *Mol. Simul.* 1:173–185. https://doi.org/10.1080/08927028808080941.

44. Moreira, R. A., H. V. Guzman, …, A. B. Poma. 2020. Characterization of structural and energetic differences between conformations of the SARS-CoV-2 spike protein. *Materials.* 13:5362.

45. Maier, J., C. Martinez, …, C. Simmerling. 2015. Ff14sb: improving the accuracy of protein side chain and backbone parameters from ff99sb. *J. Chem. Theor. Comput.* 11:3696–3713. https://doi.org/10.1021/acs.jctc.5b00255.

46. Jorgensen, W. L. 1981. Quantum and Statistical Mechanical Studies of Liquids. 10. Transferable Intermolecular Potential Functions for Water, Alcohols, and Ethers. Application to Liquid Water. *J. Am. Chem. Soc.* 103:335–340. https://doi.org/10.1021/ja00392a016.

47. Åqvist, J., P. Wennerström, …, B. O. Brandsdal. 2004. Molecular dynamics simulations of water and biomolecules with a monte carlo constant pressure algorithm. *Chem. Phys. Lett.* 384:288–294. https://www.sciencedirect.com/science/article/pii/S0009261403021687.

48. Davidchack, R. L., R. Handel, and M. V. Tretyakov. 2009. Langevin thermostat for rigid body dynamics. *J. Chem. Phys.* 130:234101. https://doi.org/10.1063/1.3149788.

49. Hopkins, C. W., S. Le Grand, …, A. E. Roitberg. 2015. Long-Time-Step Molecular Dynamics through Hydrogen Mass Repartitioning. *J. Chem. Theor. Comput.* 11:1864–1874. https://doi.org/10.1021/ct5010406.

50. Kroon, P. C., F. Grünewald, …, S. J. Marrink. 2025. Martinize2 and Vermouth provide a unified framework for molecular topology generation. *eLife.* 12:RP90627. https://doi.org/10.7554/eLife.90627.4.

51. Kabsch, W., and C. Sander. 1983. Dictionary of Protein Secondary Structure: Pattern Recognition of Hydrogen-Bonded and Geometrical Features. *Biopolymers.* 22:2577–2637. https://doi.org/10.1002/bip.360221211.

52. Bernetti, M., and G. Bussi. 2020. Pressure Control Using Stochastic Cell Rescaling. *J. Chem. Phys.* 153:114107. https://doi.org/10.1063/5.0020514.

53. Basu, A., H. Shioya, and C. Park. 2011. Statistical Inference: The Minimum Distance Approach. CRC Press. https://doi.org/10.1201/b10956.

54. Casalino, L., Z. Gaieb, …, R. E. Amaro. 2020. Beyond Shielding: The Roles of Glycans in the SARS-CoV-2 Spike Protein. *ACS Cent. Sci.* 6:1722–1734. https://doi.org/10.1021/acscentsci.0c01056.

55. Liu, S., S. Cao, …, X. Huang. 2021. Multi-level DBSCAN: A Hierarchical Density-Based Clustering Method for Analyzing Molecular Dynamics Simulation Trajectories. *bioRxiv.* https://doi.org/10.1101/2021.06.09.447666.

56. Ester, M., H. Kriegel, …, X. Xu. 1996. A Density-Based Algorithm for Discovering Clusters in Large Spatial Databases with Noise. *In* Proceedings of the Second International Conference on Knowledge Discovery and Data Mining KDD, pp. 226–231. https://doi.org/10.5555/3001460.3001507.

57. Raschka, S., J. Patterson, and C. Nolet. 2020. Machine Learning in Python: Main Developments and Technology Trends in Data Science. *Machine Learning, and Artificial Intelligence.* 11:193. https://doi.org/10.3390/info11040193.

58. Vijay-Kumar, S., C. E. Bugg, and W. J. Cook. 1987. Structure of ubiquitin refined at 1.8 Å resolution. *J. Mol. Biol.* 194:531–544. https://doi.org/10.1016/0022-2836(87)90679-6.

59. Tavares, G., P. Béguin, and P. M. Alzari. 1997. The crystal structure of a type I cohesin domain at 1.7 Å resolution. *J. Mol. Biol.* 273:701–713. https://doi.org/10.1006/jmbi.1997.1326.

60. Heo, L., and M. Feig. 2024. One bead per residue can describe all-atom protein structures. *Structure.* 32:97–111.e6. https://www.sciencedirect.com/science/article/pii/S096921262300401X.

61. Wang, L., C. Brasnett, …, S. J. Marrink. 2025. Martini3-IDP: improved Martini 3 force field for disordered proteins. *Nat. Commun.* 16:2874. https://doi.org/10.1038/s41467-025-58199-2.

Biophysical Journal, Volume *125*

Supplemental information

An optimized contact map for GōMartini 3 enabling conformational changes in protein assemblies

Gustavo E. Olivos-Ramirez, Luis F. Cofas-Vargas, Siewert J. Marrink, and Adolfo B. Poma

Supplementary Material

An optimized contact map for GōMartini 3 enabling conformational changes in protein assemblies

Gustavo E. Olivos-Ramirez[1], Luis F. Cofas-Vargas[1,2], Siewert J. Marrink[3], and Adolfo B. Poma[1,*]

[1]Department of Biosystems and Soft Matter, Institute of Fundamental Technological Research, Polish Academy of Sciences, ul. Pawińskiego 5B, 02-106, Warsaw, Poland

[2]Departamento de Química, Universidad Autónoma Metropolitana-Iztapalapa, Mexico City C.P. 09310, Mexico

[3]Groningen Biomolecular Sciences and Biotechnology Institute, University of Groningen, Nijenborgh 7, 9747 AG Groningen, The Netherlands

[*]E-mail: apoma@ippt.pan.pl

Supplementary figures

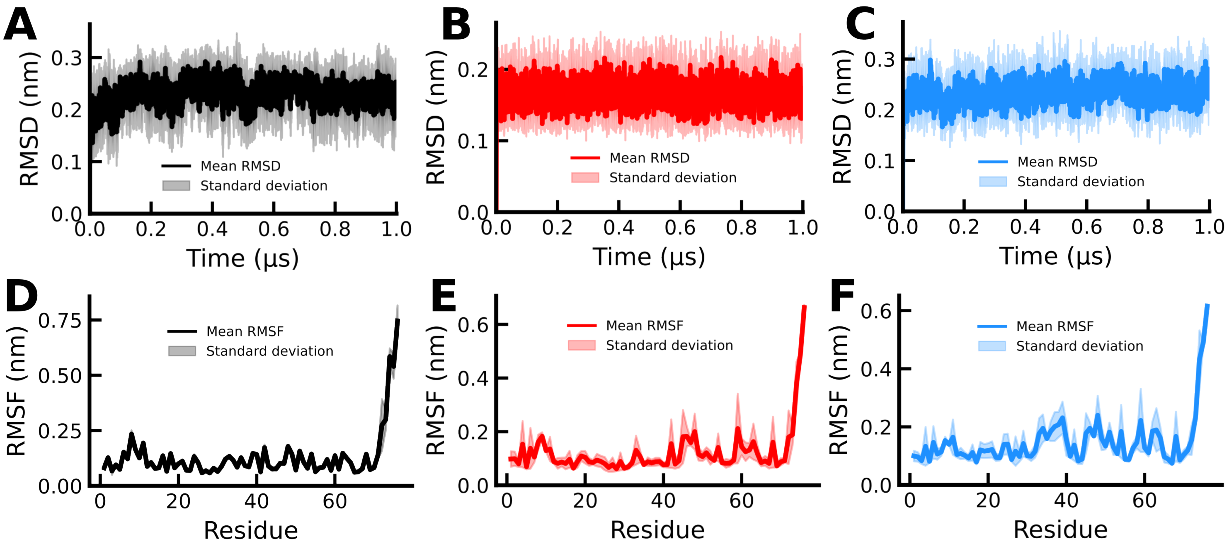

Figure S1: Time evolution of the average RMSD and RMSF of the ubiquitin protein (PDB ID: 1UBQ). Values were averaged from all simulation replicas (R1-R5). Panels A, B, C show the RMSD evolution during 1μ for AA-MD (black), GōMartini 3 with the crystal contact map (red), and GōMartini 3 with the HFC map (sky blue). Panels D, E, and F show the RMSF values for the same system.

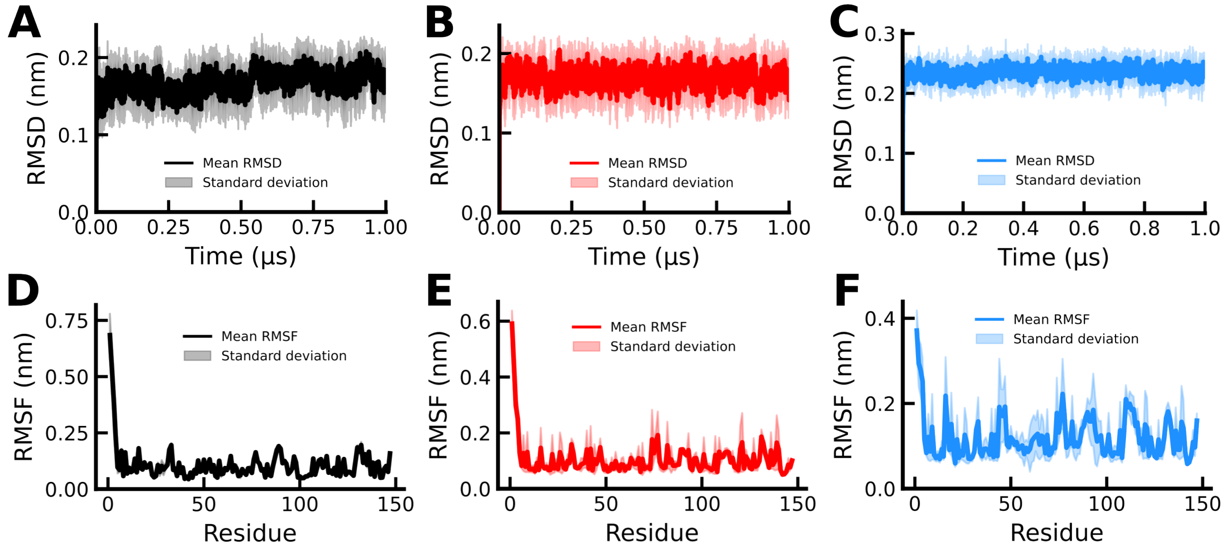

Figure S2: Time evolution of the average RMSD and RMSF of the single cohesin domain (PDB ID: 1AOH). Values were averaged from all simulation replicas (R1-R5). Panels A, B, C show the RMSD evolution during 1μ for AA-MD (black), GōMartini 3 with the crystal contact map (red), and GōMartini 3 with the HFC map (sky blue). Panels D, E, and F show the RMSF values for the same system.

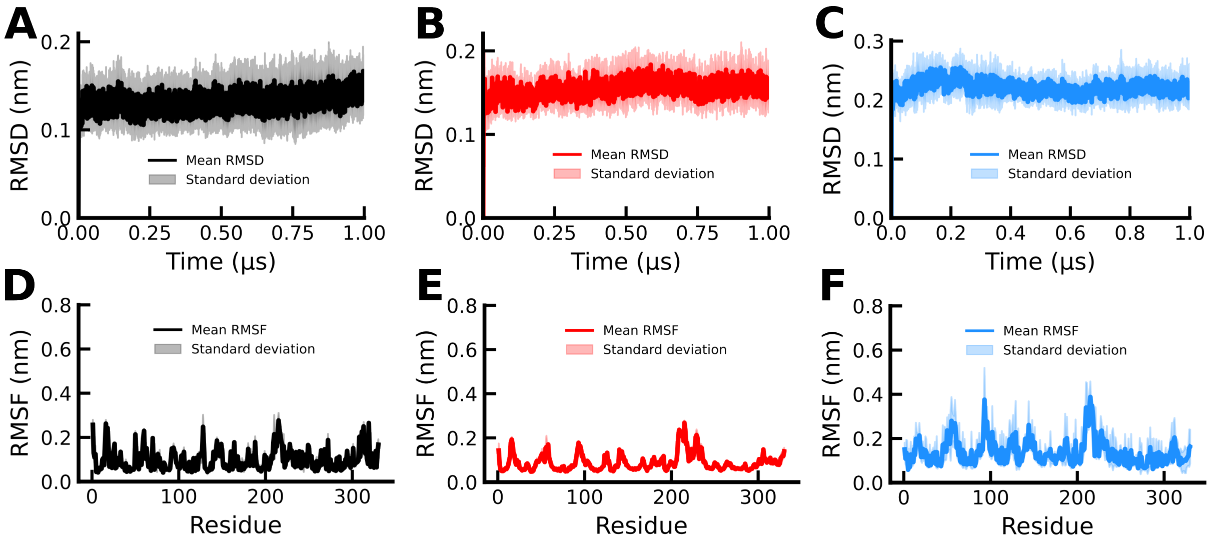

Figure S3: Time evolution of the average RMSD and RMSF of the glycoside hydrolase (PDB ID: 3W0K). Values were averaged from all simulation replicas (R1-R5). Panels A, B, C show the RMSD evolution during 1μ for AA-MD (black), GōMartini 3 with the crystal contact map (red), and GōMartini 3 with the HFC map (sky blue). Panels D, E, and F show the RMSF values for the same system.

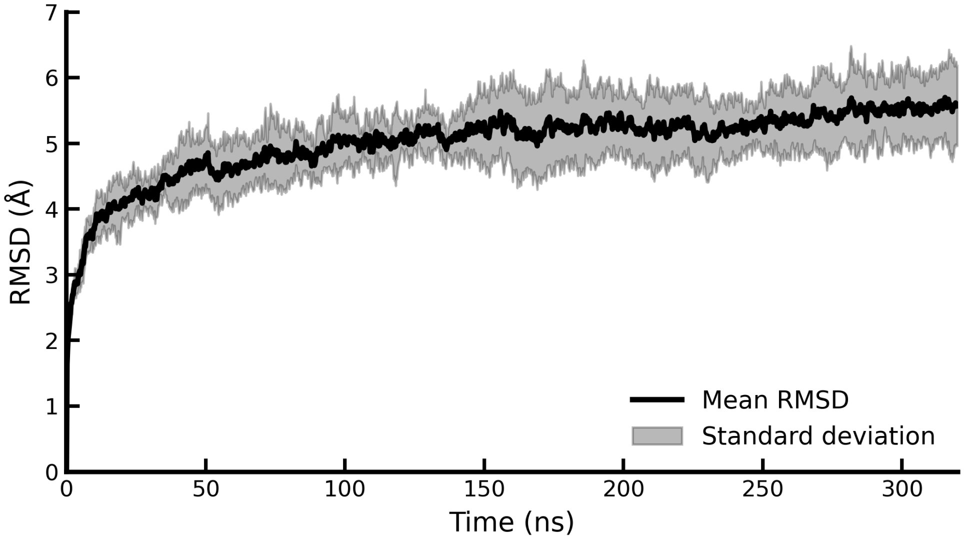

Figure S4: Time evolution of the average RMSD for all AA-MD simulation replicas (R1–R5) of the SARS-CoV-2 S protein.

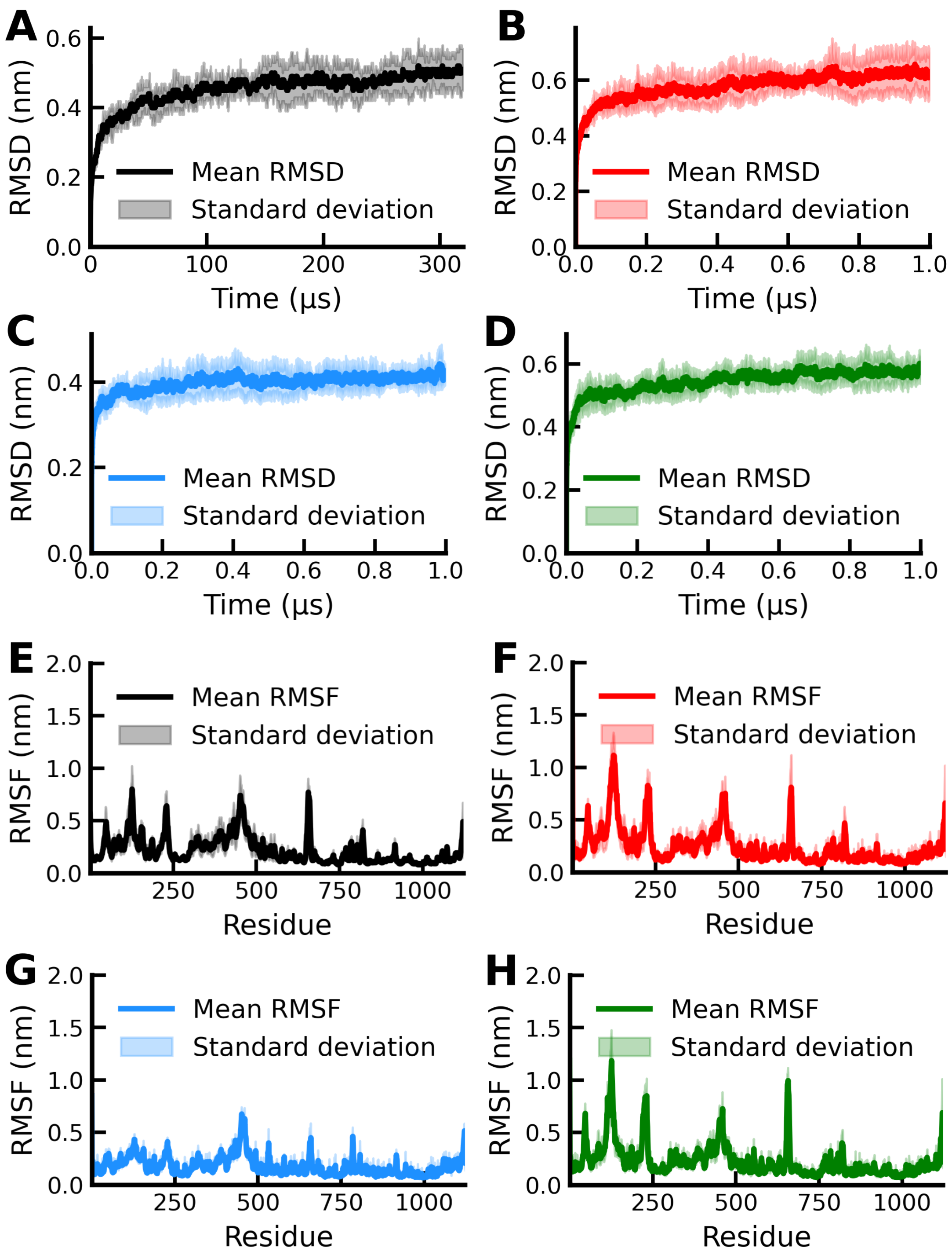

Figure S5: Time evolution of the average RMSD and RMSF for all simulation replicas (R1–R10) of the SARS-CoV-2 S protein (PDB ID: 6VSB), in AA-MD (black), GōMartini optimized-1 (red), optimized-2 (sky blue), and optimized-3 (green).

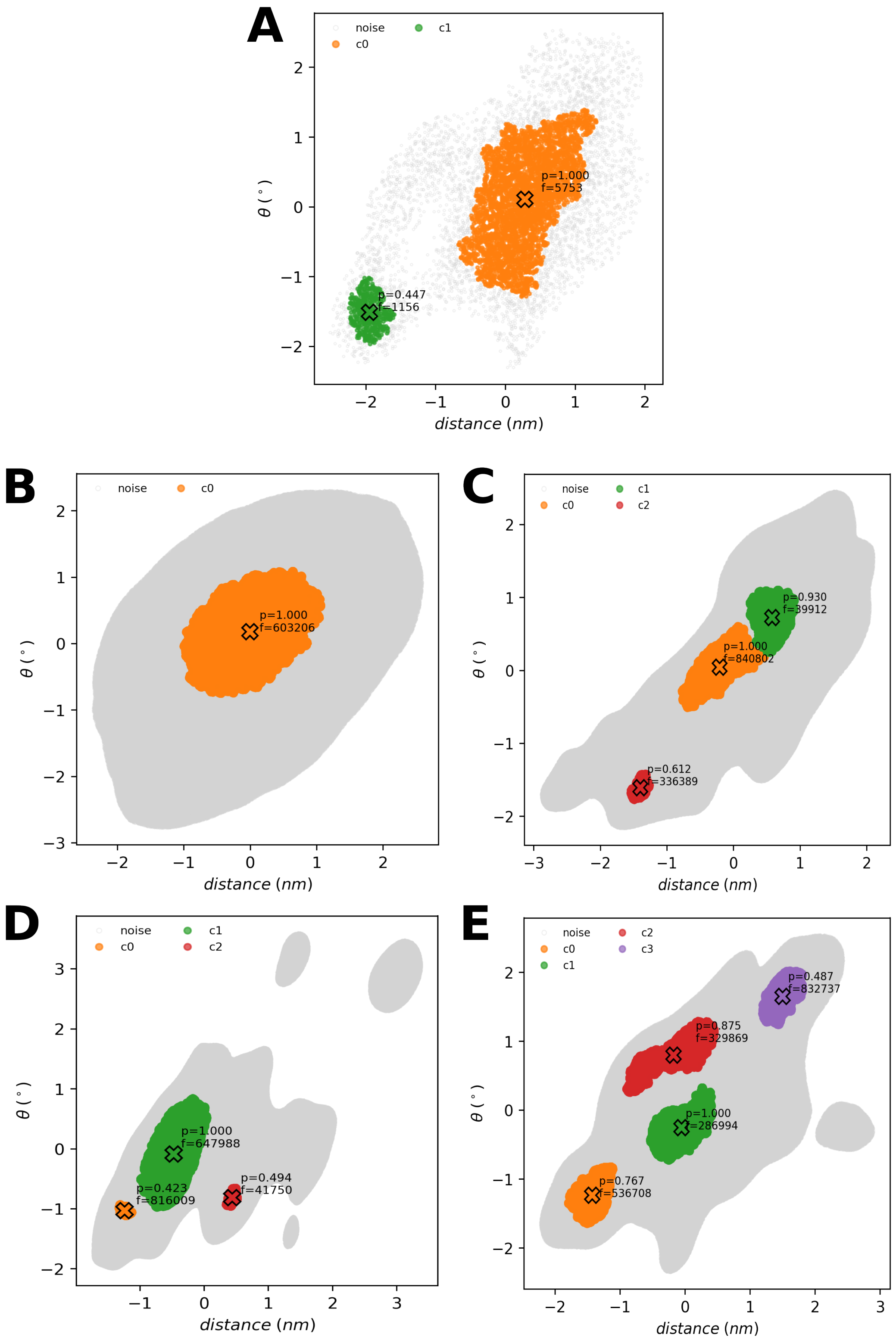

Figure S6: ML-DBSCAN of the most probable conformations of the SARS-CoV-2 S protein based on the CVs used in the FEL, from AA-MD (A), GōMartini 3 Original (B), Optimized-1 (C), Optimized-2 (D), and Optimized-3 (E) simulations.

Supplementary table

Table S1: Summary of the contact information used in each method.

Method	Contact information
AA	None
Original	NC from the initial structure
Optimized-1	HFC interchain + HFC intrachain + HFC that appear during the AA-MD
Optimized-2	HFC interchain + intrachain contacts from the reference structure
Optimized-3	HFC interchain + HFC intrachain

NC = native contacts. HFC = high-frequency contacts.

Convergence analysis of HFC protocol

To provide practical guidance for constructing robust HFC maps, we performed a systematic convergence analysis of different threshold used to define HFC. Thresholds of 0.5, 0.6, 0.7, 0.8, 0.9, and 1 were evaluated every 10 ns (from 90 to 320 ns) using the AA-MD trajectories of the S protein with five independent replicas (Figure S7 and S8). Convergence of the HFC maps was assessed with four descriptors:

(i) **Total HFC count over time.** This quantifies the temporal stabilization of the HFC set by tracking how the total number of HFC evolves as the AA-MD trajectory length increases. The emergence of a plateau indicates convergence.

(ii) **Conserved HFC fraction.** This quantifies how much of the original contact map is preserved in the HFC map by measuring (every 10 ns) the fraction of original contacts that remain present. It is computed as the number of contacts common to both maps divided by the total number of contacts in the original map.

(iii) **Newly acquired HFC.** This quantifies contacts that emerge as the AA-MD simulation progresses and are incorporated into the HFC map but are absent from the original contact map. Therefore, it provides a direct measure of how many new interactions are added beyond those present in the starting structure.

(iv) **Similarity between consecutive HFC maps (Jaccard similarity).** This quantifies how stable the HFC map is over time by comparing the contact sets obtained at consecutive time points using the Jaccard similarity. It is defined as the number of HFC shared by two maps divided by the total number of distinct contacts present in either map. We evaluated this metric from 90 to 320 ns, sampling every 10 ns. Values approaching 1 indicate that consecutive HFC maps are nearly identical, consistent with a stable HFC set.

Threshold choice should meet two important requirements: retaining enough stabilizing

contacts to preserve folding, while removing sufficient contacts from the original map to enable conformational transitions. Permissive thresholds (0.5–0.6) retain a dense network and show higher variability across time and replicas, whereas stringent thresholds (0.9–1) over-prune both conserved and newly formed contacts. Accordingly, intermediate thresholds around 0.7 provide a robust compromise, yielding reproducible contact networks (Figures S7–S8). Consistently, a stress tests at the extreme thresholds (0.5 and 1), using 1 us of CG-MD, showed that the cutoff = 1 increased RMSD relative to both cutoff = 0.5 and the AA reference. RMSF also increased globally, indicating reduced structural stability. Meanwhile, the cutoff = 0.5 stayed closer to the AA benchmark but may hinder conformational transitions due to its denser contact network (Figure S9).

Overall, this convergence workflow provides practical guidance for defining HFC maps from AA-MD trajectories and motivates the default threshold used here (0.7). As a guideline, we recommend using AA-MD segments exceeding 100 ns after initial relaxation and verifying convergence prior to HFC extraction with the same four metrics. For large multimeric assemblies, multiple independent replicas improve reproducibility and enable direct quantification of inter-replica variability. While the optimal threshold may be system-dependent, we consider a value of 0.7 to provide a robust starting point based on the observed balance between stability and conformational flexibility.

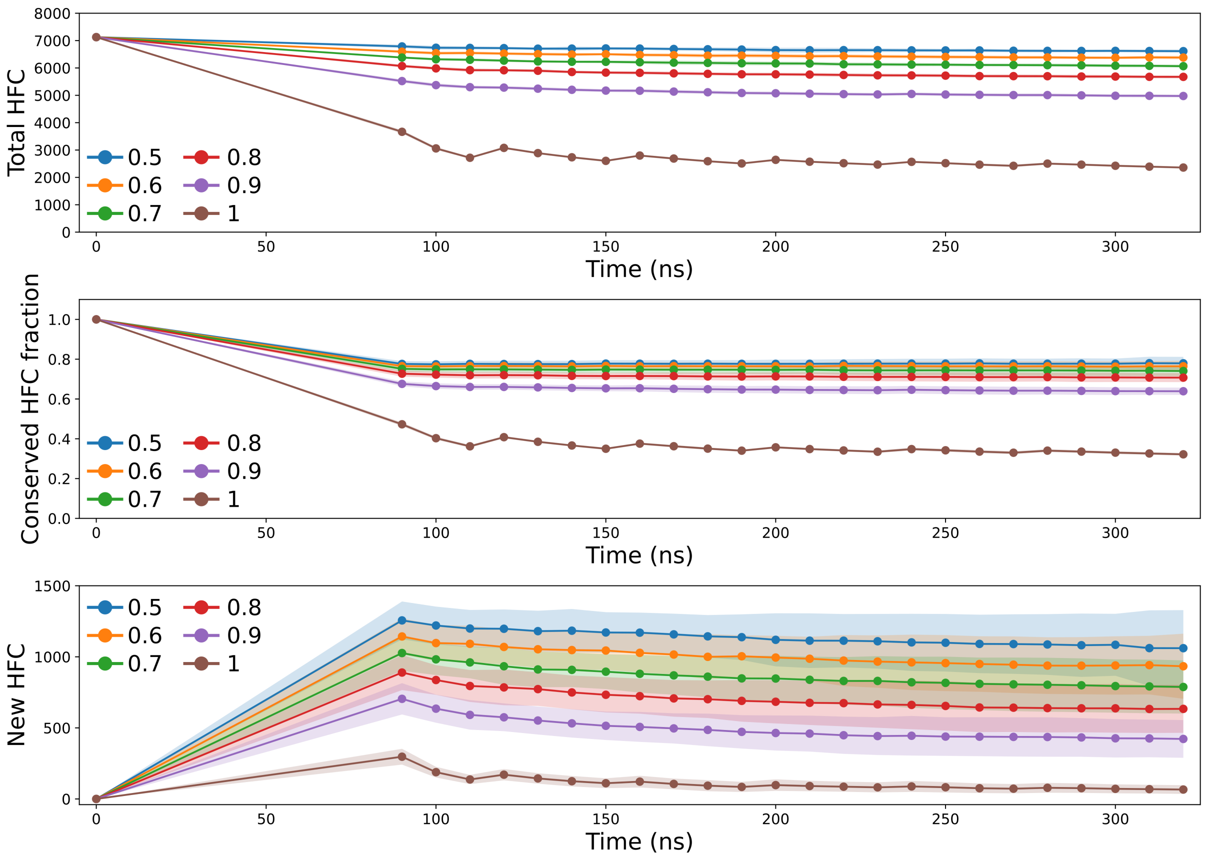

Figure S7: Convergence descriptors for HFC maps of the SARS-CoV-2 S protein computed across persistence thresholds (0.5–1) over the 90–320 ns window (10-ns stride). (A) Total number of HFC. (B) Conserved HFC ratio relative to the original contact map at $t = 0$ ns. (C) Number of newly formed HFC not present in the original map. Solid lines show the mean of each cutoff and shaded areas the SD across replicas (see Methods for definitions).

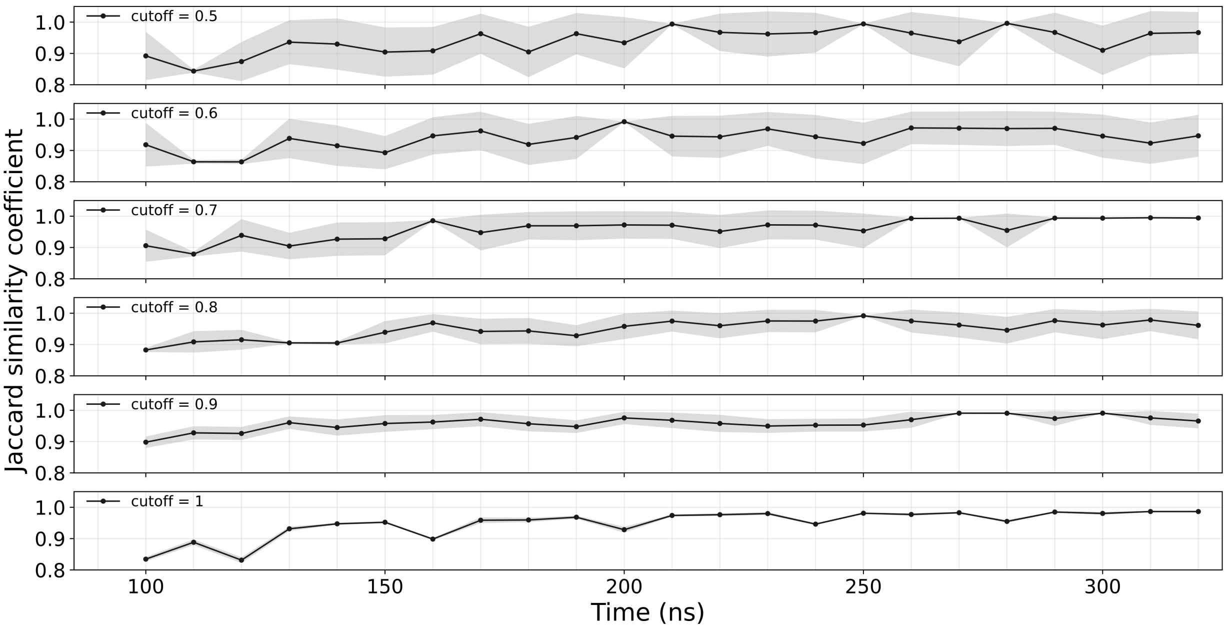

Figure S8: Jaccard similarity between consecutive HFC maps (every 10-ns) for persistence thresholds of 0.5–1 over the 90–320 ns window. Curves show the mean and shaded areas the SD across AA-MD replicas (see Methods for definition).

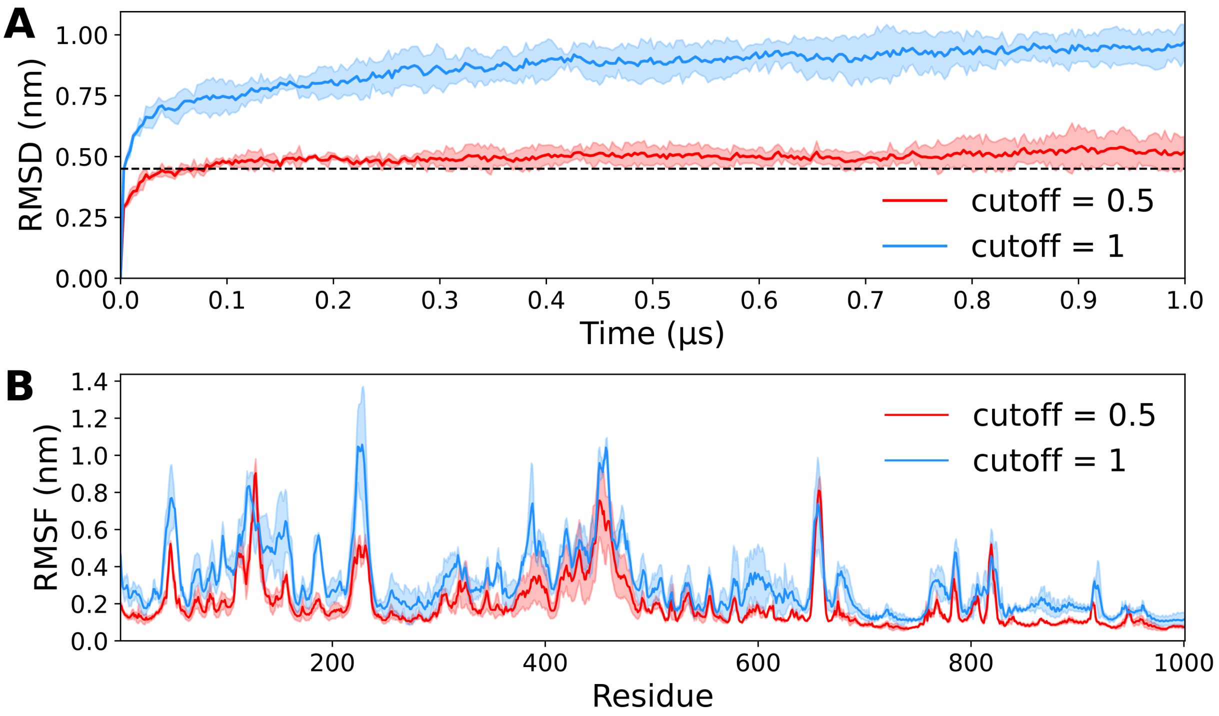

Figure S9: CG-MD simulation at extreme persistence thresholds (stress tests) for the SARS-CoV-2 S protein. (A) RMSD over 1 μs for HFC maps defined at cutoff = 0.5 and 1 (mean $\pm$ SD across three replicas); dashed line indicates the AA-MD mean RMSD. (B) Per-residue RMSF for the same conditions.
